# Total yeast and mold levels in high THC-containing cannabis (*Cannabis sativa* L.) inflorescences are influenced by genotype, environment, and pre-and post-harvest handling practices

**DOI:** 10.3389/fmicb.2023.1192035

**Published:** 2023-06-13

**Authors:** Zamir K. Punja, Li Ni, Samantha Lung, Liam Buirs

**Affiliations:** Department of Biological Sciences, Simon Fraser University, Burnaby, BC, Canada

**Keywords:** mold, marijuana, microbes, contaminants, yeasts, microbiome, quality assurance

## Abstract

Total yeast and mold (TYM) levels in inflorescences of high THC-containing *Cannabis sativa* (cannabis) are regulated to ensure that medicinal and recreational users, especially those with immunocompromised systems, are not exposed to potentially harmful levels. In North America, the limits imposed range from 1,000–10,000 cfu/g of dried product to 50,000–100,000 cfu/g, depending on the jurisdiction. Factors affecting a build-up of TYM in cannabis inflorescences have not been previously researched. In this study, >2,000 fresh and dried samples were assayed for TYM over a 3-year period (2019–2022) to identify specific factors which can contribute to TYM levels. Greenhouse-grown inflorescences were sampled before and after commercial harvest, homogenized for 30 s, and plated onto potato dextrose agar (PDA) with 140 mg/L streptomycin sulfate. Colony-forming-units (cfu) were rated after 5 days of incubation at 23°C under 10–14 h light. PDA provided more consistent counts of cfu compared to Sabouraud dextrose and tryptic soy agars. The predominant fungal genera identified by PCR of the ITS1-5.8S-ITS2 region of rDNA were *Penicillium*, *Aspergillus, Cladosporium,* and *Fusarium.* In addition, four yeast genera were recovered. In total, 21 species of fungi and yeasts constituted the total cfu present in the inflorescences. The variables that significantly (*p* < 0.05) increased these TYM levels in inflorescences were: the genotype (strain) grown, presence of leaf litter in the greenhouse, harvesting activity by workers, genotypes with a higher abundance of stigmatic tissues and inflorescence leaves, higher temperature and relative humidity within the inflorescence microclimate, time of year (May–October), method of drying buds after harvest, and inadequate drying of buds. The variables which significantly (*p* < 0.05) decreased TYM in samples were: genotypes with lower numbers of inflorescence leaves, air circulation achieved by fans during inflorescence maturation, harvesting during November–April, hang-drying of entire inflorescence stems, and drying to a moisture content of 12–14% (water activity of 0.65–0.7) or lower which was inversely correlated with cfu levels. Under these conditions, the majority of dried commercial cannabis samples contained <1,000–5,000 cfu/g. Our findings indicate that TYM in cannabis inflorescences are the result of a dynamic interaction between genotype, environment, and post-harvest handling methods. Some of these factors may be altered by cannabis producers to reduce the potential build-up of these microbes. Among the 21 fungal and yeast species recovered from greenhouse-grown cannabis inflorescences, a few could pose a potential threat to human health, while many do not and they could provide beneficial interactions within the cannabis plant. The currently recommended plating methods onto agar media and enumeration of total cfu are unable to distinguish between these two groups.

## 1. Introduction

Commercial production of drug-type *Cannabis sativa* L. (marijuana, cannabis) containing the psychoactive compound Δ^9^-tetrahydrocannabinol (also referred as Δ^9^-THC) takes place in indoor controlled environment growing spaces, in greenhouses and under field conditions. At present, a number of countries worldwide allow cannabis production for medical purposes, and some jurisdictions have approved the legal cultivation of cannabis for recreational use. The latter include countries such as Uruguay, Canada, and Thailand, as well as more than 20 individual states within the USA (Punja, 2021a). The cannabis product is derived from large complex inflorescences (racemes) comprised of aggregate pistillate structures and are harvested after 7–8 weeks of flowering under controlled photoperiods (Bernstein et al., 2019; Naim-Feil et al., 2022). The inflorescences are dried to a moisture content of 12–14% before packaging for sale (Caplan et al., 2022). All jurisdictions in which cannabis has been legally approved for medicinal and/or recreational use are required to test for the presence of total yeasts and mold (TYM) prior to sale, since these can potentially negatively affect immunocompromised patients (Jameson et al., 2022). Additional testing for specific bacterial pathogens known to affect humans, e.g., *Escherichia coli, Salmonella* spp. and *Staphylococcus aureus*, is required, in addition to testing for pesticides and mycotoxins (Boyar, 2021; Pusiak et al., 2021; Jameson et al., 2022). The microbial tests are conducted by licensed third-party laboratories and TYM presence is reported as colony-forming units per gram (cfu/g) of dried flowers (buds). The TYM limits imposed vary considerably by country and different jurisdictions, ranging from 1,000–10,000 cfu/g to higher limits of 50,000–100,000 cfu/g. In Canada, the limit is 50,000 cfu/g (Anonymous, 2018; Health Canada, 2019), while in the United States, it varies considerably by state.^1^

The results from TYM testing of cannabis samples submitted to commercial labs can vary considerably. The cfu counts may be influenced by the methods used, including the agar medium on which extracted samples are plated, the method of sample extraction, incubation time and temperature, the degree of sample dilution, and various other parameters that are discussed elsewhere (Tournas et al., 2001; Boyar, 2021; McKernan et al., 2021; Repay, 2021; Curry, 2022). In contrast, factors such as the origins of the sample, i.e., cannabis strain (genotype), growing conditions, and method of post-harvest handling, and their impact on potential TYM levels, have not been previously investigated. Sample origin is a biologically relevant variable that can potentially influence levels of TYM in the final product and is an important aspect that has been under-studied due to the complexity of identifying numerous and different variables in the cannabis growing environment and gaining access to a facility to perform such work. With the exception of DNA-based tests specifically designed to detect *Aspergillus* species, the TYM current testing does not identify specific species of fungi nor confirm presence/absence of other potentially toxigenic fungi, such as *Fusarium* and *Penicillium,* that can be present on dried cannabis (Punja, 2021b).

In this study, we sampled cannabis inflorescences in a commercial greenhouse environment at various times over a 3-year period to identify factors that could contribute to a build-up of TYM. We first describe the influence of nutrient agar media on recovery of TYM, and then compare different cannabis genotypes, time of sampling, sample source, post-harvest handling method, and extent of drying on the colony forming units of yeast and mold recovered. We also identified the most prevalent genera and species of yeast and mold recovered from these samples using molecular methods. Our findings point to cannabis genotype, production methods and post-harvest handling practices as having a significant impact on TYM in cannabis based on an analysis of >2,000 samples in this study.

## 2. Materials and methods

### 2.1. Plant materials

**Figure 1:**
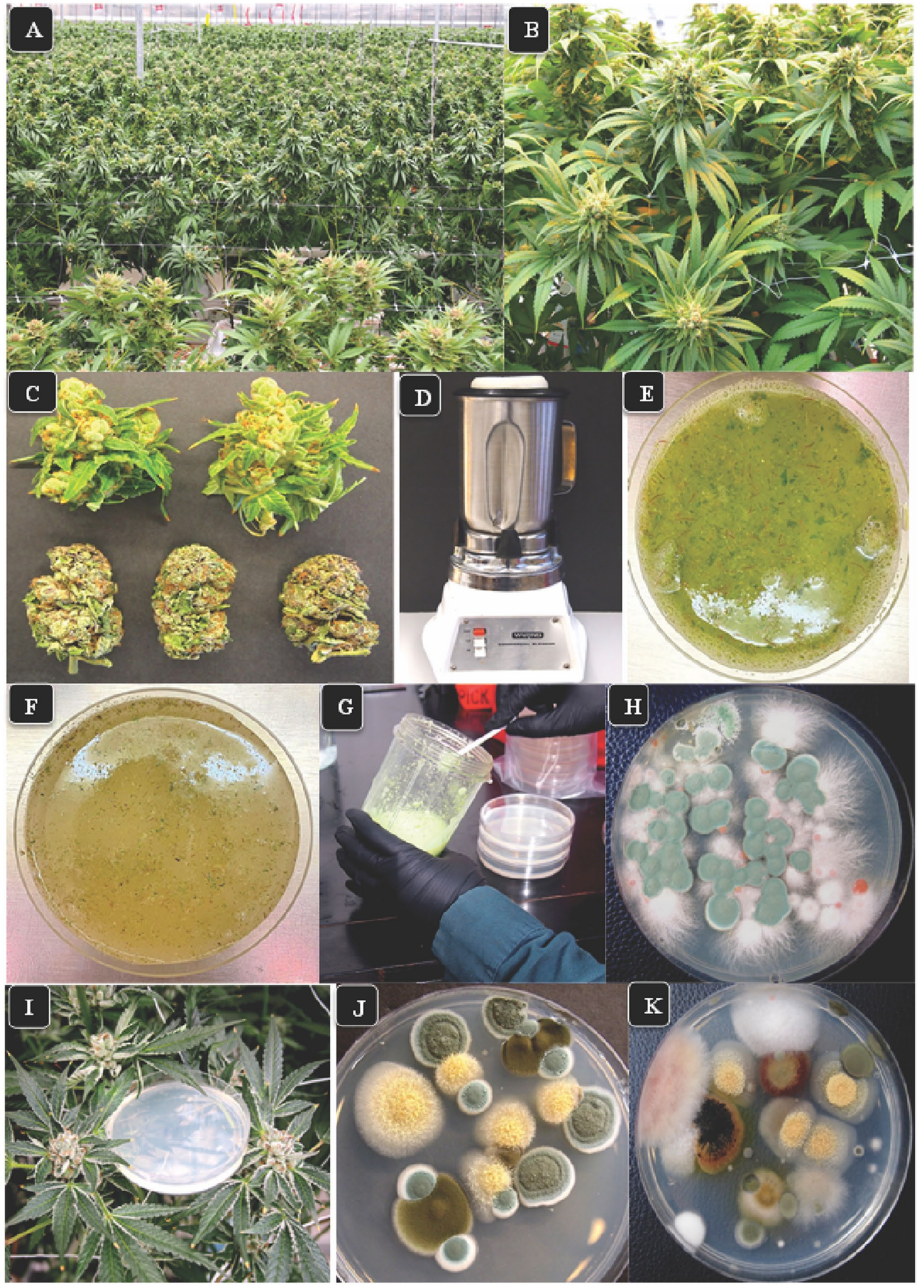
The growing environment for cannabis and various research approaches employed in this study. **(A)** Flowering plants one week prior to harvest shows the production of large numbers of inflorescences under greenhouse conditions. **(B)** A close-up of the inflorescences which are produced from multiple stems on each plant. These inflorescences were sampled for assessing total yeast and mold (TYM) in this study. **(C)** Detached fresh inflorescences (top row) and dried buds (lower row) in which the inflorescence leaves have been mechanically trimmed. **(D)** Blender used to grind tissues in water for 30 s. **(E)** A view of the homogenized tissue blend from fresh inflorescences prior to plating. **(F)** Homogenized tissue from dried inflorescences prior to plating. **(G)** Transfer slurry to Petri dishes containing agar media. **(H)** Colonies of fungi growing on PDA + S. Green colonies are *Penicillium*, pink colonies are *Fusarium*. Photo was taken 5 days after plating. **(I)** Placement of Petri dishes containing potato dextrose agar plus 140 mg/L streptomycin sulfate in the plant canopy adjacent to inflorescences to sample air-borne yeasts and molds. **(J,K)** Colonies developing on dishes exposed for 1 h in the cannabis growing environment and incubated under laboratory conditions for 5 days. The yellow colonies are *Aspergillus*, the green colonies are *Penicillium*, the brown colonies are *Cladosporium*, and the pink colony is *Fusarium*.

**Figure 2:**
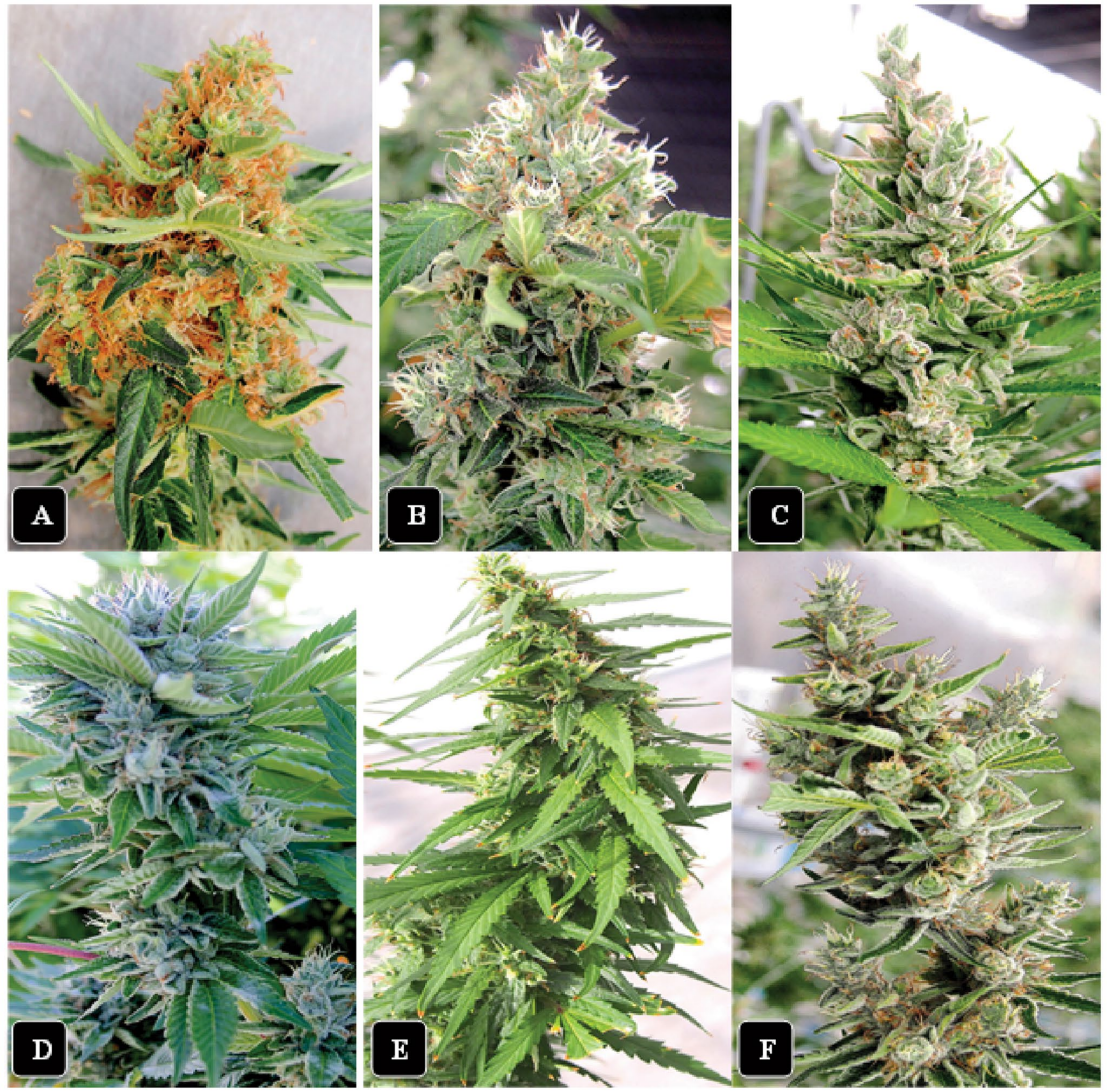
The morphological appearance of inflorescences of six cannabis genotypes included in this study at harvest time. **(A)** “Watermelon Kush”. **(B)** “Powdered Donuts”. **(C)** “Black Cherry”. **(D)** “Pink Kush”. **(E)** “Jack Herer”. **(F)** “Death Bubba”. All photos were taken at the same distance from the plant.

This research project was conducted in a Health Canada-approved licensed commercial facility in which all cannabis plants were grown using a hydroponic method of cultivation (Punja, 2021c). A range of different genotypes (strains) were grown under greenhouse conditions during the fall–winter months (October–April) and spring–summer months (May–September) over a 3-year period (2019–2022), which generally included four cropping cycles per year (Figure 1). These genotypes represented the most frequently grown at the time of sampling and are described in Table 1 and illustrated in Figure 2. During the winter period, plants were provided with supplementary lighting to achieve approximately 1,600 uEin/m^2^ of photosynthetically active radiation (PAR) using high pressure sodium lamps emitting a wavelength centered at 590 nm. During the summer period, supplementary lighting was provided where needed on overcast days to achieve the required PAR.

**Table 1:** Phenotypic characteristics of six cannabis genotypes used in the present study.

Phenotypic trait	Watermelon Kush	Pink Kush	Powdered Donuts	Jack Herer	Black Cherry	Death Bubba
Total yeast and mold levels	High	Moderate	High	Low	Moderate	Low
*Botrytis cinerea* susceptibility	High (>5% infection)	High (>5% infection)	High (>5% infection)	Low (<1% infection)	Low (<1% infection)	Low (<1% infection)
Powdery mildew susceptibility	Unknown	High	High	Unknown	Low-medium	Low
THC range	<17%	19–25%	20–28%	<17%	19–25%	17–23%
CBD range	0–1%	0–1%	0–1%	0–1%	0–1%	0–1%
Top five terpenes (highest to lowest)	Unknown	Caryophyllene Bisabolol Humulene Linalool Myrcene	Caryophyllene Myrcene Limonene Linalool Humulene	Unknown	Caryophyllene Limonene Linalool Myrcene Bisabolol	Caryophyllene Humulene Myrcene Limonene Bisabolol
Genotype characteristic	Indica	Indica	Hybrid	Sativa	Indica	Indica

All plants were initiated from vegetative cuttings taken from stock (mother) plants of various genotypes grown under greenhouse conditions with a temperature range of 23–28°C and relative humidity of 60–70%. The cuttings were dipped in a rooting powder (containing indoleacetic acid) and inserted into 2.5 cm^3^ rockwool cubes, which were then placed in trays in a propagation room under high relative humidity (80–90%) and a temperature range of 23–27°C. After 2 weeks, when cuttings had rooted, they were placed into wells cut into 10 cm^3^ rockwool blocks and transferred to a greenhouse bench for an additional 2 weeks to acclimatize and resume vegetative growth. Following this, they were placed on large cocofibre blocks, one plant per block, and transferred to a large flowering room. After 1 week of growth, the photoperiod was adjusted to 12 h lighting:12 h darkness to induce flowering (Punja, 2021a). The water and nutrient regimes were provided according to commercial growing requirements to ensure optimal growth. The plants were arranged in rows up to 104 m in length containing 216 plants, with 2 rows parallel to each other, and spacing of 0.5 m between plants. Each greenhouse compartment contained up to 27 rows of plants, which were separated by a 1 m wide walkway between rows. The plants were trained and supported by plastic mesh netting that ensured the developing inflorescences remained upright (Figures 1A,B). Pruning of leaves and training of plants were conducted manually according to commercial growing requirements as needed. Plants were grown for 7–8 weeks in the flowering period in each cropping cycle and then harvested. All stems bearing inflorescences were removed manually and the individual flower buds were detached from the stems prior to drying on racks, or the buds were detached after hang drying of the entire stem. Drying was conducted in controlled environment rooms set at 21–23°C and 50–55% relative humidity for 5–7 days.

### 2.2. Sample source

For analysis of total yeast and mold levels (TYM) in this study, all inflorescence samples were obtained from plants in the final 2 weeks of flower (usually week 7 or 8 in the flowering period) representing different genotypes being grown (fresh samples) or following drying using the rack or hang dry procedure (dried samples). Fresh samples were obtained by randomly removing the terminal inflorescences from plants within selected rows in the greenhouse and placing them inside plastic bags, which were stored overnight at 5°C prior to processing the following morning. Samples were generally collected during the morning hours (from 10:00 am to 1:00 pm). Dried samples were randomly obtained from the drying rooms, with records made of the harvest date and genotype, and stored at room temperature inside plastic bags. All samples were collected in three replicates, each from a different plant for fresh samples, and labeled according to genotype and date of sampling. Samples were collected during the winter and summer growing seasons at various times (usually weekly) during the 3-year sampling period. For data analysis to determine the influence of different variables on TYM levels, the data from replicated samples were assessed using statistical methods as described below.

### 2.3. Determining total yeast and mold levels

The tissues used for TYM analysis included fresh and dried inflorescence samples as well as dried cannabis leaves. Samples of 10 gm of fresh tissue or 1 gm of dried tissues were homogenized in a Waring blender to which 100 mL of sterile distilled water was added and the mixture was blended at high speed for 30 s. A volume of 400 mL of water was added, stirred, and an aliquot of 0.5 mL was transferred to agar media and spread using a Q-tip. Three agar media were compared for frequency of recovery of yeasts and molds, colony characteristics, and uniformity across replicate dishes. These media included potato dextrose agar (Sigma-Aldrich Canada, Oakville, ON, Canada) to which 140 mg/L of streptomycin sulfate was added to inhibit bacterial growth (PDA + S), Sabouraud dextrose agar (Fisher Scientific Company, Ottawa, ON, Canada) and tryptic soy agar (Sigma-Aldrich, Oakville, ON, Canada). Duplicate samples of each tissue source were used for grinding and each sample was plated onto four replicate Petri dishes of each of the different media types (*n* = 8). One batch of dishes was incubated at ambient room temperature (21–23°C) and a duplicate set was placed in an incubator set at 28 ± 0.5°C and both sets were provided with 10–14 h/day of florescent lighting, to replicate flower room conditions. After 5 days, the Petri dishes were assessed for the total number of colonies and the size and morphology of the colonies were recorded. This procedure was repeated for multiple samples of cannabis collected at different times to ensure the consistency of the results. The mean number of colonies in each sample on each of the different media was determined and data are presented with the standard error of the mean (SE, *n* = 8). Morphological unique colonies were transferred to fresh dishes of PDA + S and following growth over 2 weeks, sub-cultured again to ensure purity, and then subjected to molecular identification as described below.

To compare the accuracy of the grinding and plating assay used in this study with analyses conducted by commercial laboratories that routinely conduct TYM assays, a replicated set of fresh and dried flower samples were subdivided into three sub-samples; two were sent to individual commercial laboratories located in Canada (their identity is kept anonymous). The third replicate was processed using the method described above. TYM counts per g of sample were compared for the three sub-samples. The process was repeated for dried leaf material that was collected from the greenhouse floor.

### 2.4. Morphological and molecular identification of TYM

To identify each morphologically unique colony to genus and species, a PCR method utilizing primers for the ITS1-5.8S-ITS2 region of ribosomal DNA (rDNA) was used (Punja, 2021b). DNA was extracted from mycelium scraped from the surface of colonies on PDA + S using the QIAGEN DNeasy Plant Mini Kit. Aliquots of 1 μL containing 5–20 ng DNA were used for PCR in a 25 μL reaction volume consisting of 2.5 μL 10X buffer (containing 15 mM MgCl_2_), 0.5 μL 10 mM dNTP, 0.25 μL Taq DNA Polymerase (QIAGEN), 0.25 μL 10 mM forward and reverse primers, as well as 20.25 μL DNAse- and RNAse-free water (Invitrogen). The universal eukaryotic primers UN-UP18 S42 (5′-CGTAACAAGGTTTCCGTAGGTGAAC-3′) and UN-LO28 S576B (5′-GTTTCTTTTCCTCC GCTTATTAATATG-3′) were used (Punja, 2021b,c). All PCR amplifications were performed in a MyCycler thermocycler (BIORAD) with the following program: 3 min at 94°C; 30 s at 94°C, 30 s at 60°C, 3 min at 72°C (35 cycles); and 7 min at 72°C. PCR products were separated on 1% agarose gels and bands of the expected size (*ca.* 700 bp) were purified with QIAquick Gel Extraction Kit and sent to Eurofins Genomics (Eurofins MWG Operon LLC 2016, Louisville, KY) for sequencing. The resulting sequences were compared to the corresponding ITS1-ITS2 sequences from the National Centre for Biotechnology Information (NCBI) GenBank database to confirm species identity using only sequence identity values above 99%. A total of 450 fungal and yeast colonies were analyzed. Genbank accession numbers only for the unique fungal species recovered are presented in Supplementary Figure 1.

### 2.5. Air sampling for yeasts and molds

Air sampling was conducted in the greenhouse environment by placing Petri dishes containing PDA + S close to the inflorescences on plants where they were left with lids removed for 60 min (Figure 1). The sampling was mostly conducted between 11:00 a.m. and 2:00 p.m. The lids were replaced and the dishes were taken to the laboratory for identification of morphologically distinct colonies of fungal species as described above. A total of 500 Petri dishes were used at various times over the duration of this study to characterize the air-borne yeasts and molds. More than 200 individual colonies were selected and analyzed by PCR.

### 2.6. Influence of cannabis genotypes on TYM levels

To assess the potential influence of six cannabis genotypes on the presence of TYM in the inflorescences, fresh inflorescence samples were obtained and homogenized and plated at various times as described previously. To determine the influence of time of the year on populations of TYM, replicate samples from the six genotypes were collected from plants growing in June, July, August, and September of 2021 from sequentially harvested crops and plated onto PDA + S. Comparisons were made of the total number of colonies and types of fungi and yeast recovered from combined replicate samples of each genotype (*n* = 16).

To further determine the possible role of inflorescence leaves on TYM, five genotypes were selected for study. These were “Watermelon Kush”, “Powdered Donuts”, “Death Bubba”, “Pink Kush” and “Jack Herer” (genotype “Black Cherry” was unavailable). Terminal inflorescence stems measuring up to 15 cm in height were removed from the plants and brought back to the laboratory to be analyzed. All leaves emerging from the inflorescences, which included large multi-foliate fan leaves and single unifoliate leaves, were carefully dissected using a scalpel and counted from five replicate samples. To determine the TYM levels in these tissues, 10 g of fan leaves or unifoliate leaves were homogenized and plated onto PDA + S. Colony counts were made after 5 days of incubation (*n* = 20). The experiment was conducted three times. The mean number of inflorescence leaves in each genotype and the TYM colonies derived from each sample were averaged and expressed with the standard error of the mean (SE, *n* = 20). To determine if surface-sterilization of inflorescence leaves had an impact on the TYM recovered, dissected leaves from three genotypes were immersed in 0.625% NaOCl for 1 min followed by 70% EtOH for 30 s and then rinsed in sterile distilled water. Samples of 10 g were ground and plated onto PDA + S as previously described. Colony counts were obtained after 5 days of incubation.

To observe the extent to which fungi growing on inflorescences were able to colonize stigmatic surfaces and inflorescence leaves, samples of “Watermelon Kush” and “Powdered Donuts” were harvested from these plants. The latter genotype was visibly colonized by *Fusarium sporotrichiodes* that grew within the inflorescence tissues from naturally derived inoculum. Tissue segments were prepared for scanning microscopy according to the method described by Punja et al. (2023).

### 2.7. Temperature and relative humidity measurements within inflorescences

A hand-held temperature and humidity recording psychrometer (Reed 8,706 psychrometer, Reed Instruments, Newmarket, ON, Canada) was used to measure temperature and relative humidity within intact inflorescences on cannabis plants that were approaching harvest. The probe was gently inserted into the inflorescence tissues and held in place for 10 s to obtain a reading. Similar measurements were made of the surrounding environment by exposing the probe to ambient greenhouse conditions adjacent to the row of plants. Temperature and humidity measurements were recorded daily over a 2-week period during June 2021 at four different times: 6:45 a.m., 10:45 a.m., 2:45 p.m., and 6:45 p.m. Cannabis genotype “Pink Kush” was used for these measurements. Additional measurements were made at these four times over a 72-h period for two additional genotypes: “Powdered Donuts” and “Death Bubba” and compared to ambient conditions.

To investigate the influence of circulating air movement on temperature and relative humidity measurements in inflorescences, several fans were mounted above cannabis plants on central posts in the greenhouse and left on for 24 h daily over a 7-day period prior to harvest. Measurements of temperature and relative humidity were made in inflorescences of “Pink Kush” and “Powdered Donuts” under the fans, as well as on control plants not exposed to circulating air in adjacent rows of plants. The trial was conducted during August 2021 and repeated once during January 2022. Inflorescence samples (*n* = 5) were collected from plants grown with and without fans and TYM levels were assessed by plating as described previously.

### 2.8. Effects of post-harvest handling methods on TYM levels

Following harvest of cannabis inflorescences, the individual flowers (buds) may be detached from the main stem using a machine that physically strips them off (a process called bucking or destemming) (Caplan et al., 2022), after which the inflorescence leaves are trimmed in a machine that passes the buds through a series of rotating blades (wet trim). The processed buds are dried on a rack on trays placed in a drying room (the wet trim, rack dry method) (Caplan et al., 2022). Alternatively, the entire stem is harvested and hung upside down in the drying room, and following drying, removal of buds from stems and trimming is conducted as for wet trim (hang dry method) (Caplan et al., 2022). To determine TYM levels in inflorescences that were processed by wet trim vs. hang dry, samples (total of 35) were obtained at multiple times from the same greenhouse compartment representing different genotypes to allow a comparison to be made of the resulting impact on TYM. The data from four representative samples are presented.

To estimate the impact of drying on moisture loss and TYM levels, samples of hang-dry inflorescences were taken at daily intervals over a 6-day period from a drying room. Samples were collected from 12 locations within the room and the moisture content was estimated using the method following U.S. Pharmacopeia USP 731.^2^

Water activity measurements were also made for each sample following the procedure of USP 922.^3^ TYM levels were assessed in each sample as described previously (*n* = 12). For each sample, four replicate Petri dishes were used and colonies were counted after 5 days of incubation at 23°C. The procedure was repeated three times using different batches of cannabis samples. The mean number of colonies for each sample was determined and expressed with the standard error of the mean (SE, *n* = 12).

Inflorescence samples of genotype “Pink Kush” and “Powdered Donuts” processed using the hang dry method were sent to a commercial laboratory for estimation of TYM over a 12-month period from October 2020 to September 2021, representing samples from different cropping cycles. The data from a total of 110 samples were grouped according to the reported TYM levels, which ranged from <100 to >50,000 cfu/g. Because these were individual samples obtained at various time points during the 12-month sampling period, there was no replicated data available for statistical analysis. The individual data were plotted according to frequency of samples present in each TYM category to allow comparisons to be made.

### 2.9. Statistical analyses

To assess whether there were significant differences among genotypes, inflorescence leaf sterilization methods, impact of air circulation, and drying methods, on TYM levels, statistical analysis was done using RStudio Version 1.3.1093. An analysis of variance (ANOVA) was used to determine significance between the means of TYM from replicated and repeated experiments in these studies. Tukey’s *post-hoc* test was used to determine which variables were significantly different (*p* < 0.05) from one another.

## 3. Results

### 3.1. Sampling procedures

The greenhouse environment and development of cannabis plants sampled in this study is shown in Figures 1A,B. The plants were grown in parallel rows and individually spaced about 0.5 m apart. By the time of harvest, the foliage of plants had intermingled, and the inflorescences were fully developed (Figure 1B). Each inflorescence produced multi-foliate fan leaves at the bottom with unifoliate inflorescence leaves emerging at various positions toward the top. A comparison of a fresh cannabis sample with inflorescence leaves and a dried sample in which the inflorescence leaves had been mechanically trimmed is shown in Figure 1C. These tissues were homogenized (Figure 1D) to produce a suspension that was green in color when taken from a fresh sample (Figure 1E) or brown when originating from a dried sample (Figure 1F). The resulting extract (0.5 mL) was plated onto Petri dishes containing PDA + S and spread out using a Q-tip (Figure 1G). A range of fungal colonies could be seen growing after a 5-day incubation period at 23°C (Figure 1H). The air sampling method for detecting yeasts and molds involved placing Petri dishes containing PDA + S on the plant canopy adjacent to the inflorescences at various locations in the greenhouse (Figure 1I). The colonies that emerged were numerous and diverse in morphology (Figures 1J,K) and were counted after 5 days and identified to species level using morphological and molecular methods.

### 3.2. Comparative morphology of cannabis genotypes

Six genotypes were grown in the same greenhouse compartment and were sampled at the same time to allow a direct comparison to be made under similar growing conditions (Figure 2). These genotypes differed in several phenotypic features that included the overall size of the inflorescences produced (height and width), density of pistils produced (as indicated by the exposed stigmatic surfaces visible on the surface), degree of compactness of the inflorescence (tight vs. loosely structured) and extent of inflorescence leaves formed (unifoliate leaves emerging directly from the inflorescence) (Bernstein et al., 2019). They also differed in their susceptibility to several common diseases (Table 1). There were no differences in the range of THC or CBD levels or presence of the six most common terpenes among the genotypes. However, a correlation was observed between the relative levels of TYM present in inflorescences and the susceptibility of the same genotypes to *Botrytis* bud rot (Mahmoud et al., 2023) and powdery mildew infection (Scott and Punja, 2022) i.e., higher susceptibility to these diseases was associated with higher TYM levels in the same genotypes under similar growing conditions.

### 3.3. Determining total yeast and mold levels

**Figure 3:**
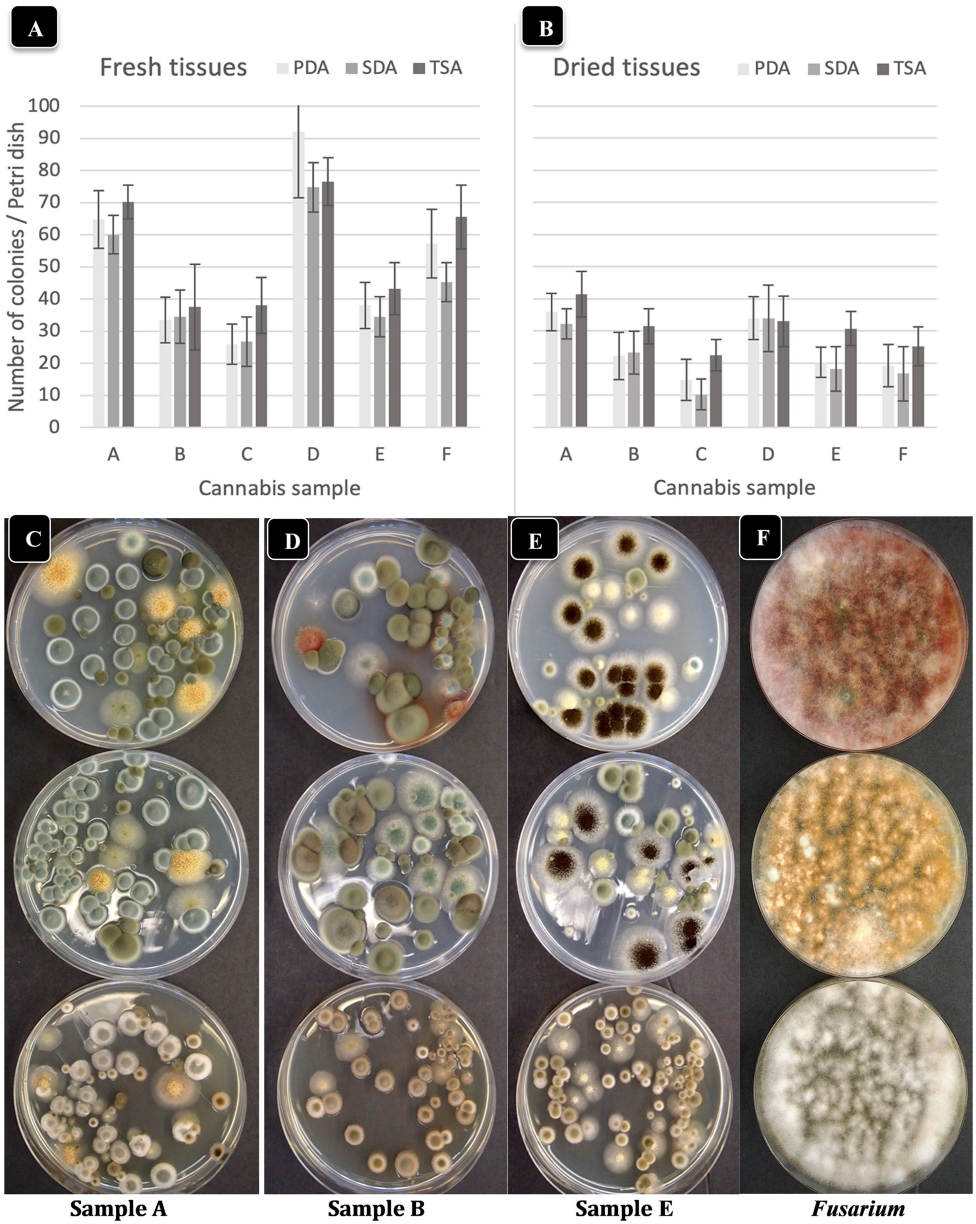
Comparison of three agar media for the recovery of yeasts and molds originating from fresh and dried cannabis samples. The three media tested were PDA (potato dextrose agar containing 140 mg/L streptomycin sulfate), SDA (Sabouraud dextrose agar) and TSA (tryptic soy agar). The same sample source was used for the fresh and dried tissue analysis. **(A,B)** The results from six cannabis samples are shown, where each sample is represented by a different plant source and/or genotype. The samples were run in duplicate and the extracts were plated onto 4 Petri dishes (*n* = 8) for each sample. The number of colonies per Petri dish was rated after 5 days of incubation at 23°C. The vertical bars represent means for each sample with standard error bars reflecting 95% confidence intervals around the mean. Dried tissues showed consistently lower colony numbers compared to fresh samples. **(C–F)** The morphological appearance of colonies of yeasts and molds on the three different media tested. Results from samples **(A,B,E)** are shown. In each row, the top dish contains PDA + S, the middle dish contains SDA and the bottom dish contains TSA. While colony numbers were similar across all media, morphological differences between colonies of different species were most clearly seen on PDA + S. Colonies of TSA were much smaller in size compared to the other two media. In the far right panel, *Fusarium* was plated out to show the more distinct colony color and morphology on PDA + S compared to the other two media.

The total number of colonies of yeast and mold present in six fresh and dried cannabis samples plated onto three different media is shown in Figures 3A,B. The standard error bars from four replicates and two duplicates of each sample showed the variation (SE) around the mean. While samples differed in the initial levels of TYM present, the different media showed similar trends. There were consistently lower TYM levels in dried samples compared to fresh samples (Figure 3B). The morphological appearance of colonies on the three-agar media after 5 days of incubation are compared in Figures 3C–E for samples identified as A, B, and E. Colonies on PDA and SDA displayed vibrant colony colors not seen on TSA. On the latter medium, colonies were mostly beige-brown in color and were indistinguishable from each other morphologically. On PDA and SDA, colonies could be identified by colony colors: green (*Penicillium*), yellow (*Aspergillus*), black (*Aspergillus*), and olive-brown (*Cladosporium*). These identifications were confirmed by PCR analysis (see below). A culture of *Fusarium graminearum* was plated on all three media for comparison of morphological appearance. On PDA, the colony produced a characteristic red color, which appeared yellow on SDA and white on TSA (Figure 3F). Based on these results, PDA + S was selected as the preferred medium to be included in this study for ease of identification and enumeration of colonies.

**Figure 4:**
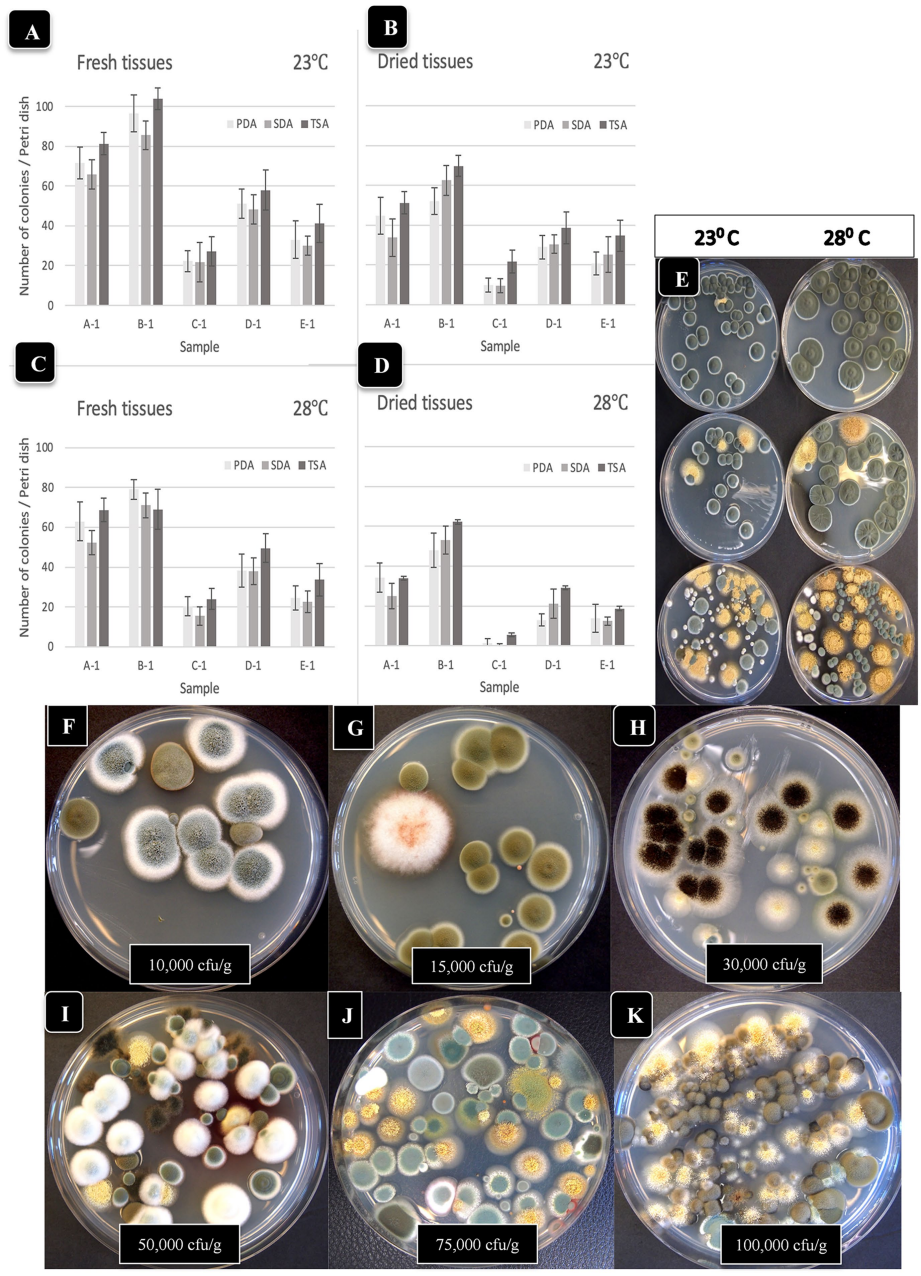
Comparison of the effect of two temperatures (23°C, 28°C) on the recovery of yeasts and molds originating from fresh and dried cannabis samples. Tissues were homogenized in a blender and plated onto each of 3 media (PDA + S, SDA and TSA) and colony numbers were rated after 5 days of incubation at each temperature. The samples were run in duplicate and the extracts were plated onto 4 Petri dishes (*n* = 8) of each medium for each sample. **(A,B)** Colony numbers recovered at 23°C from fresh and dried tissues. **(C,D)** Colony numbers recovered at 28°C from fresh and dried tissues. The vertical bars represent means with standard error bars showing variation around the mean. **(E)** Comparison of colony growth on PDA + S at two temperatures for 3 tissue samples. **(F–K)** The range of colony numbers observed in this study on PDA + S, ranging from 10,000 cfu/g to 100,000 cfu/g after 5 days of incubation at 23°C.

To investigate the effect of incubation temperature (23°C vs. 28°C) on TYM growth, duplicate sets of four dishes containing the three media which received extracts from homogenized fresh and dried cannabis tissues were incubated for 5 days and compared for colony numbers and visual appearance of the colonies. Colony size was greater at 28°C vs. 23°C, but overall colony number was generally lower at 28°C (Figures 4A–D). Dried tissues consistently had lower TYM levels compared to fresh tissues, as noted previously. The larger colony sizes which intermingled with each other can be seen on dishes incubated at 28°C (Figure 4E). This made it difficult to discern individual colonies compared to incubation at 23°C, which was used as the preferred incubation temperature throughout the study.

To calculate the levels of TYM present in samples (expressed as cfu/g of tissue), the amount of tissue weighed out (fresh or dried) and the dilution factor was used. Fresh tissue (10 g) or dried tissue (1 g, assumed to contain 10% moisture) were both added to 100 mL of water for blending. Following the addition of 400 mL of water and subsequent plating of 0.5 mL onto Petri dishes, the dilution factor was determined to be 1,000-fold for both fresh and dried samples. Therefore, a single colony represented 1,000 cfu/g while 10 colonies represented 10,000 cfu/g. The range of colonies observed in samples included in this study was <10,000 cfu/g (Figure 4F) to 15,000–100,000 (Figures 4G–K). The colonies were diverse in appearance and following PCR of the ITS1-5.8S-ITS2 region of rDNA, were identified primarily as *Penicillium* (green or whitish green colonies, Figure 4F), *Cladosporium* (olive-brown colonies, Figure 4G), *Aspergillus* spp. (black colonies [Figure 4H], or yellow colonies [Figure 4I], or olive-green colonies [Figure 4J]).

### 3.4. Morphological appearance of TYM colonies

**Figure 5:**
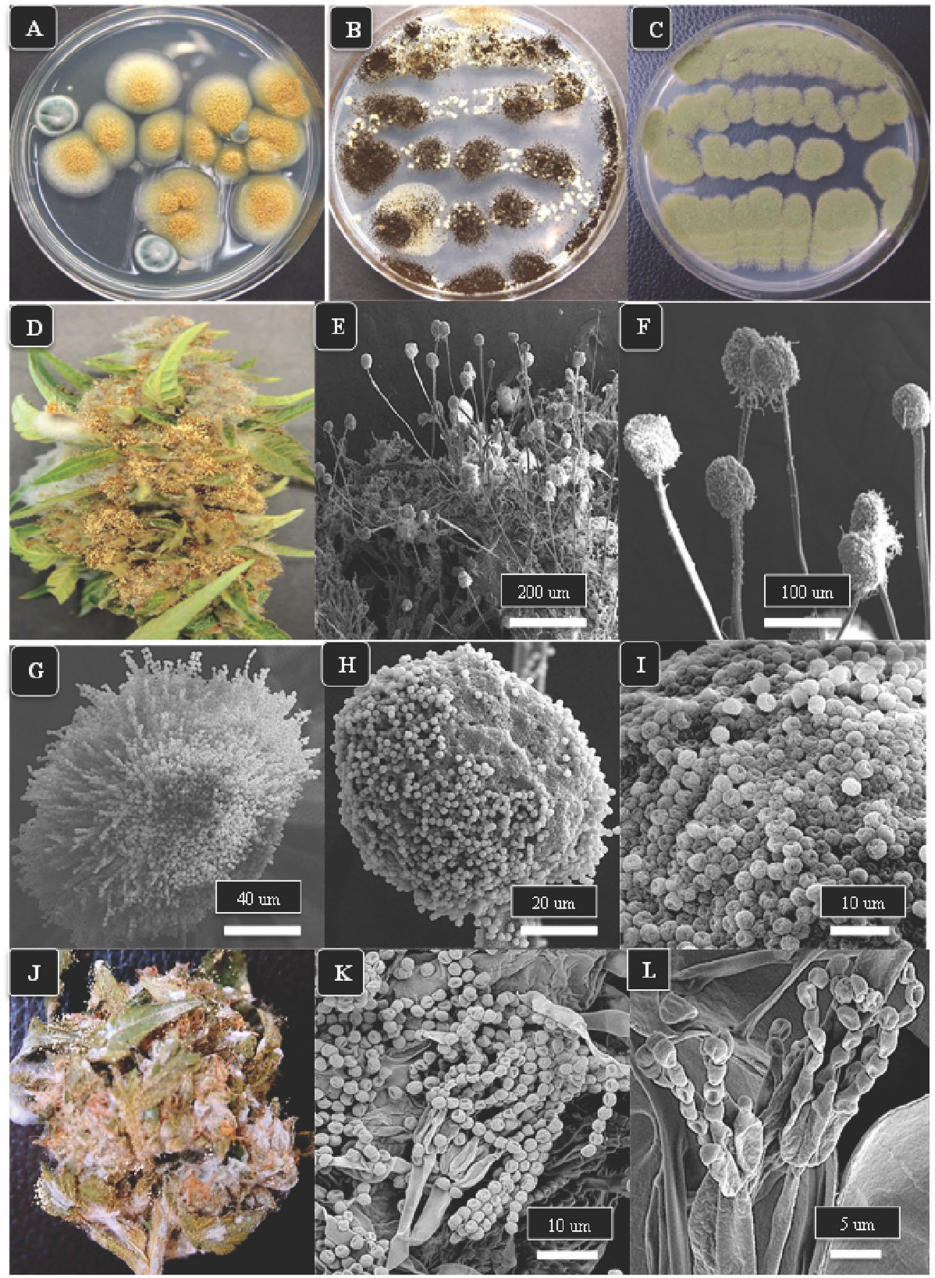
The appearance of colonies of three *Aspergillus* species and *Penicillium* sp. and scanning electron microscopic observations of spore production. **(A–C)** Colonies of *A. ochraceus*, *A. niger* and *A. flavus*, respectively, after 5 days of growth on PDA + S. **(D)** *Aspergillus ochraceus* emerging from a cannabis inflorescence incubated under high humidity conditions for 3 days. **(E,F)** The spore-bearing structures (conidiophores) of *Aspergillus* consist of stalks upon which are produced a mass of spores formed in chains, as seen in **(G–I)**. **(J)** Growth of *Penicillium* species on the surface of a cannabis inflorescence kept under high humidity for 5 days appears as a whitish-blue mycelial growth. **(K,L)** Spores of *Penicillium* sp. are produced in chains at the tips of conidiophores and are produced from structures called phialides (arrow).

A description of *Aspergillus* and *Penicillium* colonies originating from cannabis inflorescences and their spore structures as viewed in the light and scanning electron microscopes are shown in Figure 5. On PDA + S agar, *Aspergillus ochraceus*, *A. niger*, and *A. flavus* produced sufficiently distinct colonies on PDA + S that they could be identified using colony morphology in subsequent tissue plating analyses (Figures 5A–C). *Aspergillus ochraceus* was the most common species recovered in this study, appearing bright yellow on cannabis inflorescences when placed under high humidity conditions for 48 h (Figure 5D). This species, as well as all other *Aspergillus* species, produced spores borne in clusters (heads) on erect conidiophores (stalks) that could be seen using the scanning electron microscope (Figures 5E,F). The spore surface was spiny (echinulate) under high magnification (Figures 5G–I). In contrast, *Penicillium* species growing on cannabis inflorescences appeared whitish-blue (Figure 5J) and the spores were similarly produced in long chains on conidiophores and the spore surface was spiny (Figures 5K,L). In addition to these fungi, a number of yeasts were also recovered from cannabis tissues and are shown in Supplementary Figure 1.

### 3.5. Air sampling for yeasts and molds

**Figure 6:**
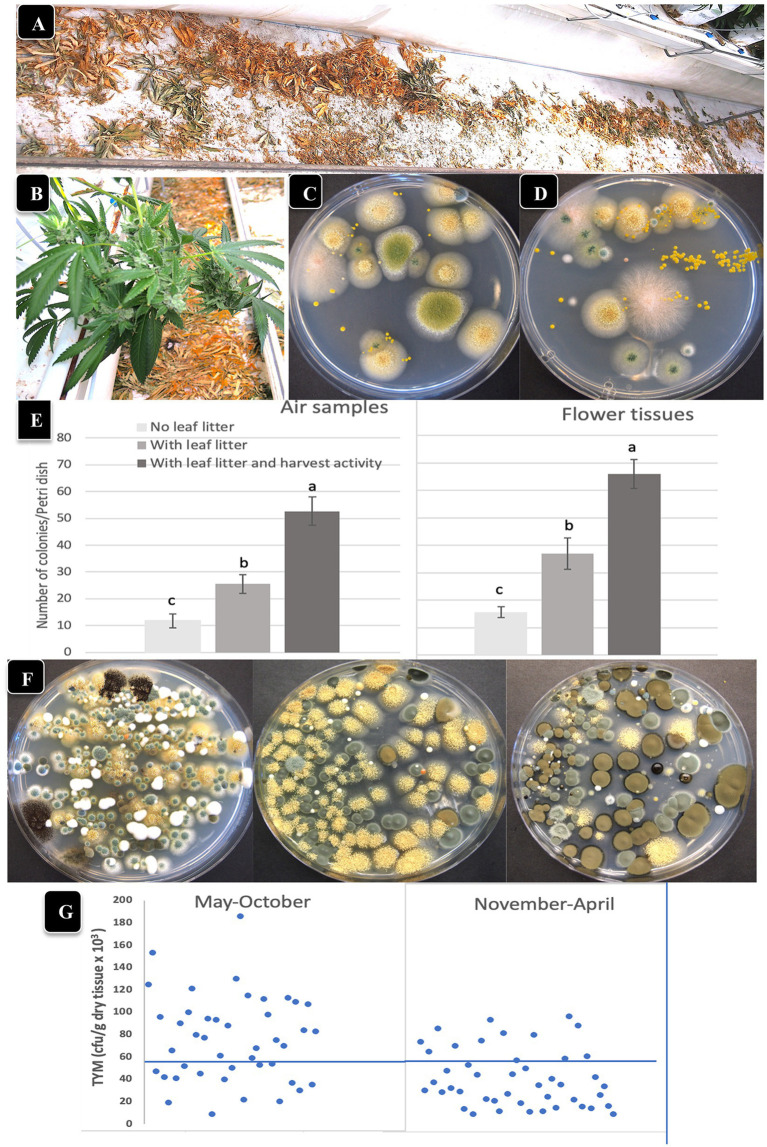
The impact of leaf litter left on the greenhouse floor on total yeast and mold levels present in the air and on adjacent cannabis inflorescences. **(A,B)** Leaf litter was purposely left on the floor to dry below cannabis plants. **(C,D)** Grinding and plating samples of leaf litter onto PDA + S reveals the presence of *Aspergillus*, *Fusarium* and *Penicillium*. **(E)** Comparison of TYM levels in air samples (left) and on cannabis inflorescences (right) in the absence and presence of leaf litter, and in the presence of leaf litter with harvesting activity by workers. Vertical bars show the mean of 20 Petri dishes with error bars reflecting 95% confidence intervals. Letters above bars denote significant differences according to Tukey’s *post-hoc* test (*p* < 0.05). **(F)** Petri dishes with colonies of TYM exceeding 100,000 cfu/gm originating from cannabis inflorescences exposed to leaf litter and harvesting activities. Yellow colonies are *Aspergillus ochraceus*, dark brown colonies are *Cladosporium cladosporiodies*, and blue-green colonies are *Penicillium* spp. **(G)** Comparison of TYM levels in cannabis inflorescences in fresh samples harvested during the period May–October versus November–April. A greater number of samples exceeded the threshold limit, indicated by the horizontal line at 50,000 cfu/g, in the May–October period.

Air sampling Petri dishes were used to determine the effect of leaf litter presence/absence and harvesting activity on TYM levels in the air and on inflorescences. Leaves that were removed during pruning activities were deliberately left on the greenhouse floor to dry over the 7–8 week flowering period in certain locations (Figures 6A,B), and compared to adjacent areas with no leaf litter. Inflorescences were harvested from both areas and analyzed for TYM. When these dried leaf tissues were homogenized and plated (1 g dry weight) to determine the background microbes present in them, developing colonies were identified as *A. ochraceus*, *A. fumigatus, Penicillium*, and *Fusarium* sp. (pink colonies; Figures 6C,D). The impact of harvesting activity, defined as the activity of workers that removed the mesh netting around plants prior to cutting and removing the inflorescence stems, as well as stepping on the dried leaves on the floor where present, on TYM in the air and on flowers, was also assessed. The data show that both the presence of leaf litter as well as the harvesting activity contributed significantly (*p* < 0.05) to increasing the TYM levels in the air and on flower tissues compared to where no leaf litter was present (Figures 6E,F). The Petri dishes from these studies frequently contained >100,000 cfu/g of tissue and consisted mostly of *Penicillium*, *Aspergillus* and *Cladosporium* species (Figure 6F). There appeared to be a seasonal effect on the TYM levels in these leaf litter experiments. When conducted during May–October compared to November–April (Figure 6G), there were more samples that exceeded the 50,000 cfu/g level in the former. These data were compiled from samples collected at various times and from different genotypes and are presented as a composite within the two sampling periods indicated.

### 3.6. Influence of cannabis genotypes on TYM levels

**Figure 7:**
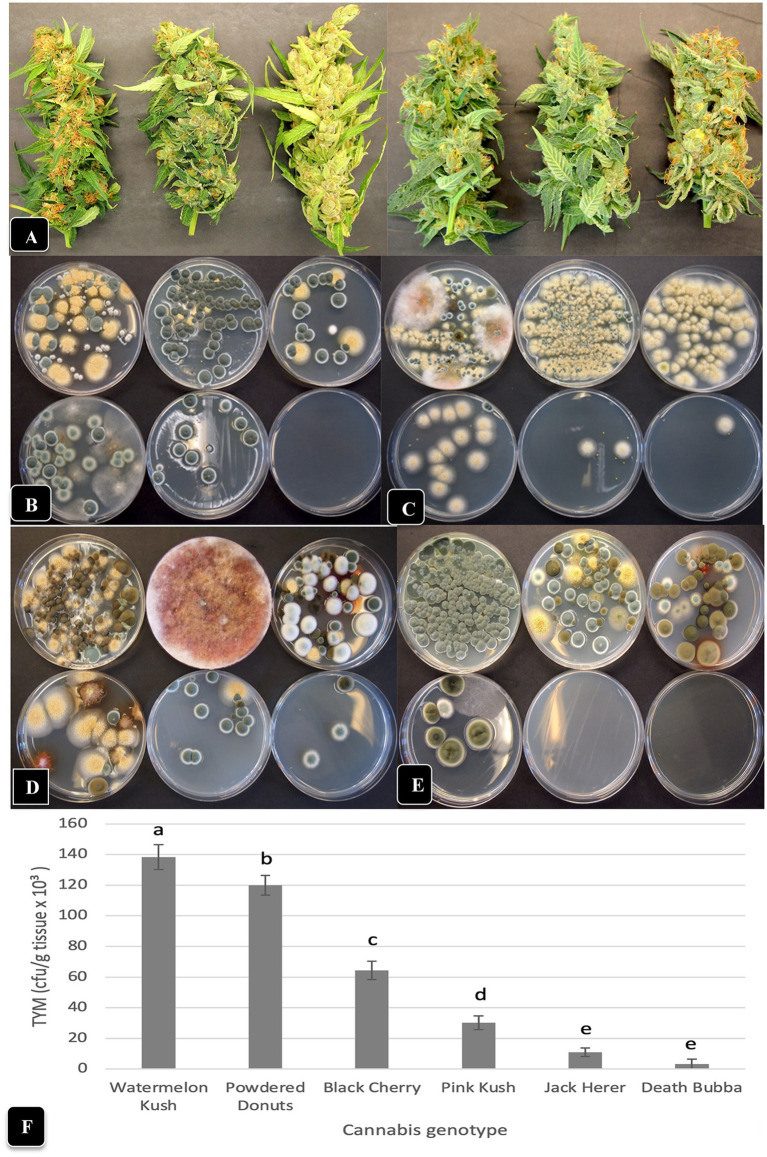
Comparison of TYM in fresh cannabis inflorescences of six different genotypes grown under the same conditions and harvested over a 4-month period (June–September). **(A)** The appearance of inflorescence samples, going from left to right, are: “Watermelon Kush”, “Powdered Donuts”, “Black Cherry”, “Pink Kush”, “Jack Herer” and “Death Bubba”. **(B–E)** The number and diversity of colonies of TYM on Petri dishes that correspond to the above genotypes, when sampled in the months of **(B)** June, **(C)** July, **(D)** August and **(E)** September. The yellow colonies are *Aspergillus ochraceus*, the blue-green colonies are *Penicillium* spp., the dark brown colonies are *Cladosporium cladosporiodes*, and the pinkish-red colonies are *Fusarium sporotrichiodes*. Photos were taken after 5 days of incubation of Petri dishes at 23°C. Note the very low or complete absence of colonies from inflorescences of “Jack Herer” and “Death Bubba”. **(F)** Average TYM levels in inflorescences of six cannabis genotypes shown in **(A)**. Data are the means from replicate dishes (*n* = 16) from the 4 months of sampling. Error bars show 95% confidence intervals and vertical bars with different letters are significantly different from each other according to Tukey’s *post-hoc* test (*p* < 0.05).

The comparative morphology and visual appearance of the inflorescence samples from six cannabis genotypes included in this study are shown in Figure 7A. When these inflorescence tissues were homogenized and plated during the months of June, July, August and September, the six genotypes were shown to contain significantly (*p* < 0.05) different numbers of TYM (Figure 7F) as well as diverse fungal morphologies (Figures 7B–E). In all months sampled, the most prevalent fungi were *Penicillium* spp., *A. ochraceus*, and *Cladosporium* spp. based on the morphology of the colonies, which was subsequently confirmed by PCR. In addition, bright pinkish-red colonies of *Fusarium sporotrichiodes* were seen in July and August (Figures 7C,D). The genotypes “Jack Herer” and “Death Bubba” consistently had the lowest TYM during all months of sampling (p < 0.05) (Figure 7F). The highest levels were seen on “Watermelon Kush” and “Powdered Donuts”.

### 3.7. Influence of inflorescence leaves of cannabis genotypes on TYM

**Figure 8:**
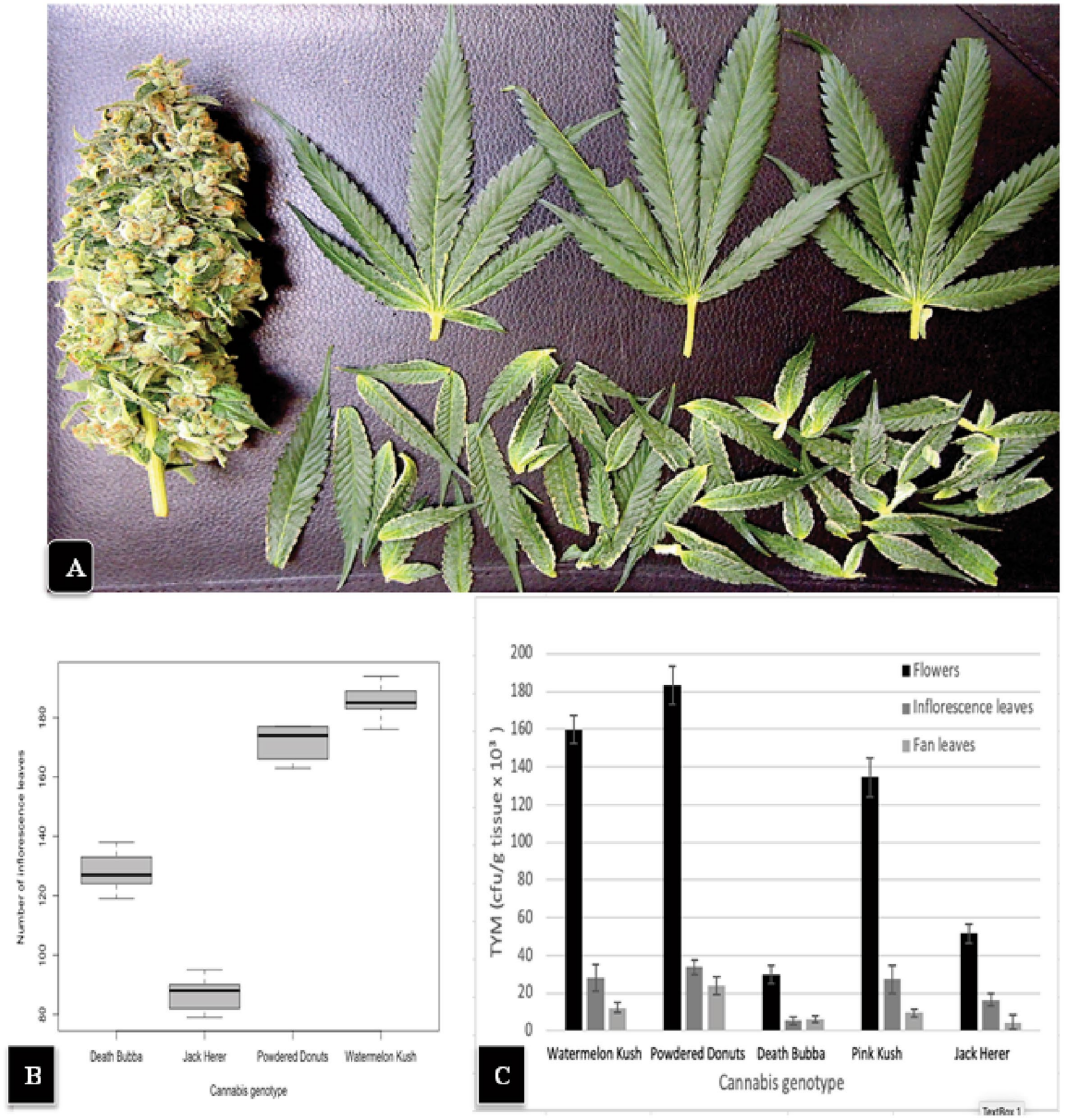
**(A)** A hand-trimmed inflorescence of “Powdered Donuts” (left) showing the large number of smaller inflorescence leaves that were removed (bottom) and 3 large fans leaves (top) originating from the inflorescence structure. **(B)** A comparison of the average number of inflorescence leaves in 4 cannabis genotypes. Note the significantly larger numbers in “Watermelon Kush” and “Powdered Donuts” compared to “Death Bubba” and “Jack Herer”. The box-plot graph shows the variance around the mean with standard error bars reflecting the 95% confidence intervals. **(C)** Levels of TYM present in fan leaves, inflorescence leaves, and the trimmed flower structure of 5 cannabis genotypes. Standard error bars reflect 95% confidence intervals.

The general appearance of a large mature inflorescence of cannabis consists of a central stalk around which aggregates of pistillate structures are produced to form a raceme, with larger 3 or 5-foliate fan leaves and smaller unifoliate inflorescence leaves emerging from all sides (Figure 2). To determine the total number of inflorescence leaves produced by different cannabis genotypes, inflorescences of genotypes “Watermelon Kush”, “Powdered Donuts”, “Jack Herer” and “Death Bubba” were obtained from greenhouse-grown plants and brought back to the laboratory. Careful dissection was performed to remove all attached leaves using a scalpel and pair of forceps. An example of a dissected inflorescence of “Powdered Donuts” and dissected leaves laid out is shown in Figure 8A. The inflorescence leaves were enumerated and the results are presented in Figure 8B. They show that there were significant differences (*p* < 0.05) among the four genotypes in the number of inflorescence leaves, with “Powdered Donuts” and “Watermelon Kush” having more compared to “Jack Herer” and “Death Bubba”. Following this, the fan leaves, inflorescence leaves and remaining inflorescence tissues were homogenized and plated onto PDA + S to determine TYM levels in each tissue type. The results (Figure 8C) show that the majority of TYM were associated with the inflorescence tissues and much fewer numbers were found in the inflorescence leaves and fan leaves. Genotypes “Watermelon Kush” and “Powdered Donuts” had the highest TYM (Figure 8C).

**Figure 9:**
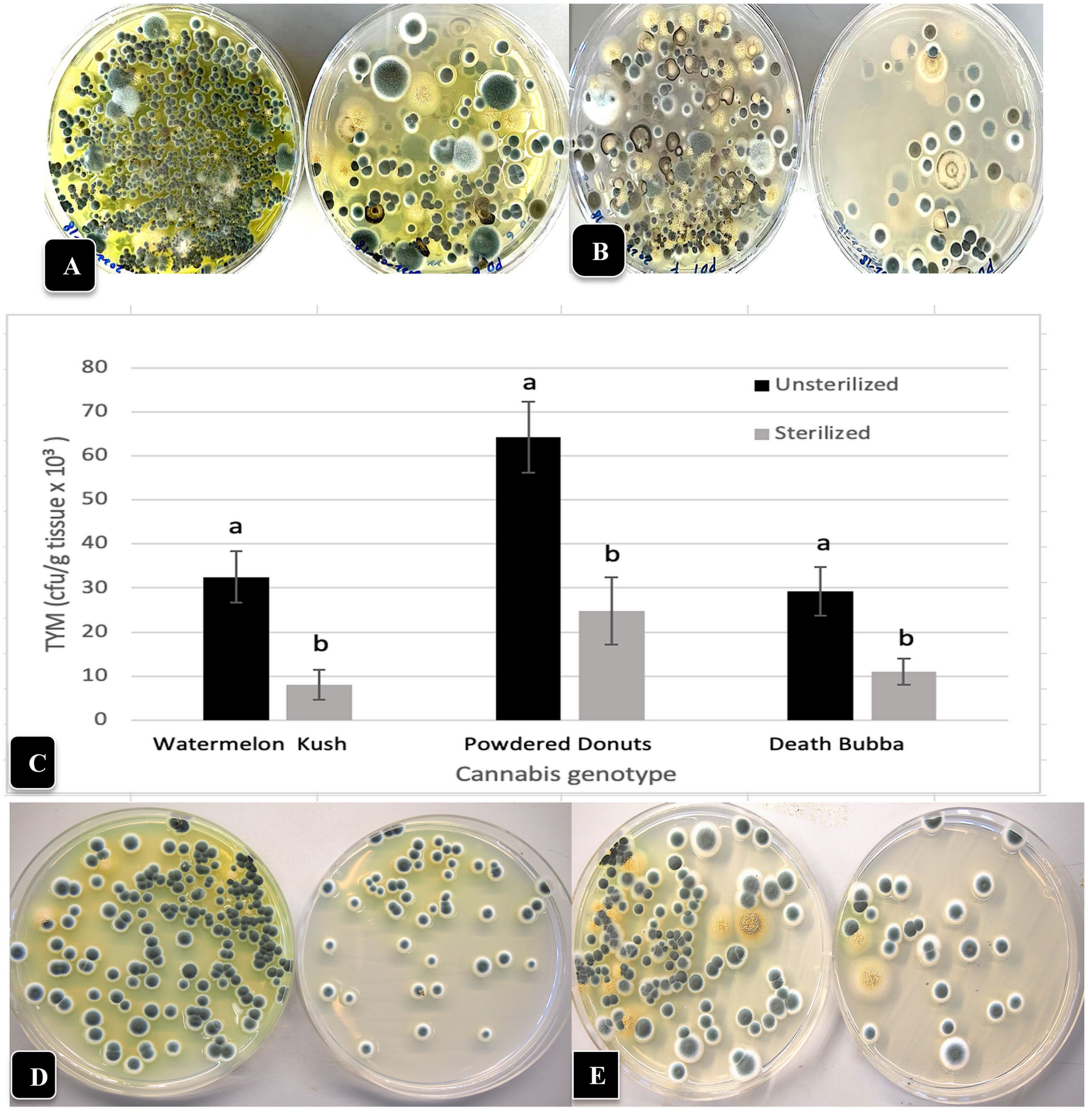
The TYM levels present in inflorescence flower tissues and inflorescence leaves following dissection of the inflorescence to remove the inflorescence leaves in two cannabis genotypes. **(A)** Colonies on PDA + S originating from “Watermelon Kush” inflorescences (left dish) compared to inflorescence leaves (right dish) shows a much higher level of TYM in the flower tissues but inflorescence leaves still contain a significant level of TYM. **(B)** Same as **(A)** but the genotype is “Powdered Donuts” showing the same trend. **(C)** The effect of surface-sterilization using a combination of bleach and ethanol on TYM in cannabis inflorescence leaves from 3 genotypes. Data are the means from replicates (*n* = 20). Error bars show 95% confidence intervals and vertical bars with different letters are significantly different from each other according to Tukey’s *post-hoc* test (*p* < 0.05). **(D,E)** Comparison of levels of TYM in inflorescence leaves of 2 cannabis genotypes without surface-sterilization (left Petri dish) and following sterilization (right Petri dish). **(D)** Genotype “Watermelon Kush”. **(E)** Genotype “Powdered Donuts”.

A comparison of the TYM present in inflorescence flower tissues compared to inflorescence leaves of genotypes “Watermelon Kush” and “Powdered Donuts” when plated onto PDA + S is shown in Figures 9A,B. Considerably more colonies were observed on the inflorescence flower tissues compared to the inflorescence leaves in both genotypes. When the inflorescence leaves were surface-sterilized, the TYM levels were significantly reduced in the three genotypes tested but a range of 10,000–20,000 cfu/g could still be recovered (Figure 9C). A comparison of the Petri dishes showing colonies from sterilized and unsterilized tissues from “Watermelon Kush” and “Powdered Donuts” is shown in Figures 9D,E.

### 3.8. Temperature and relative humidity measurements within inflorescences

**Figure 10:**
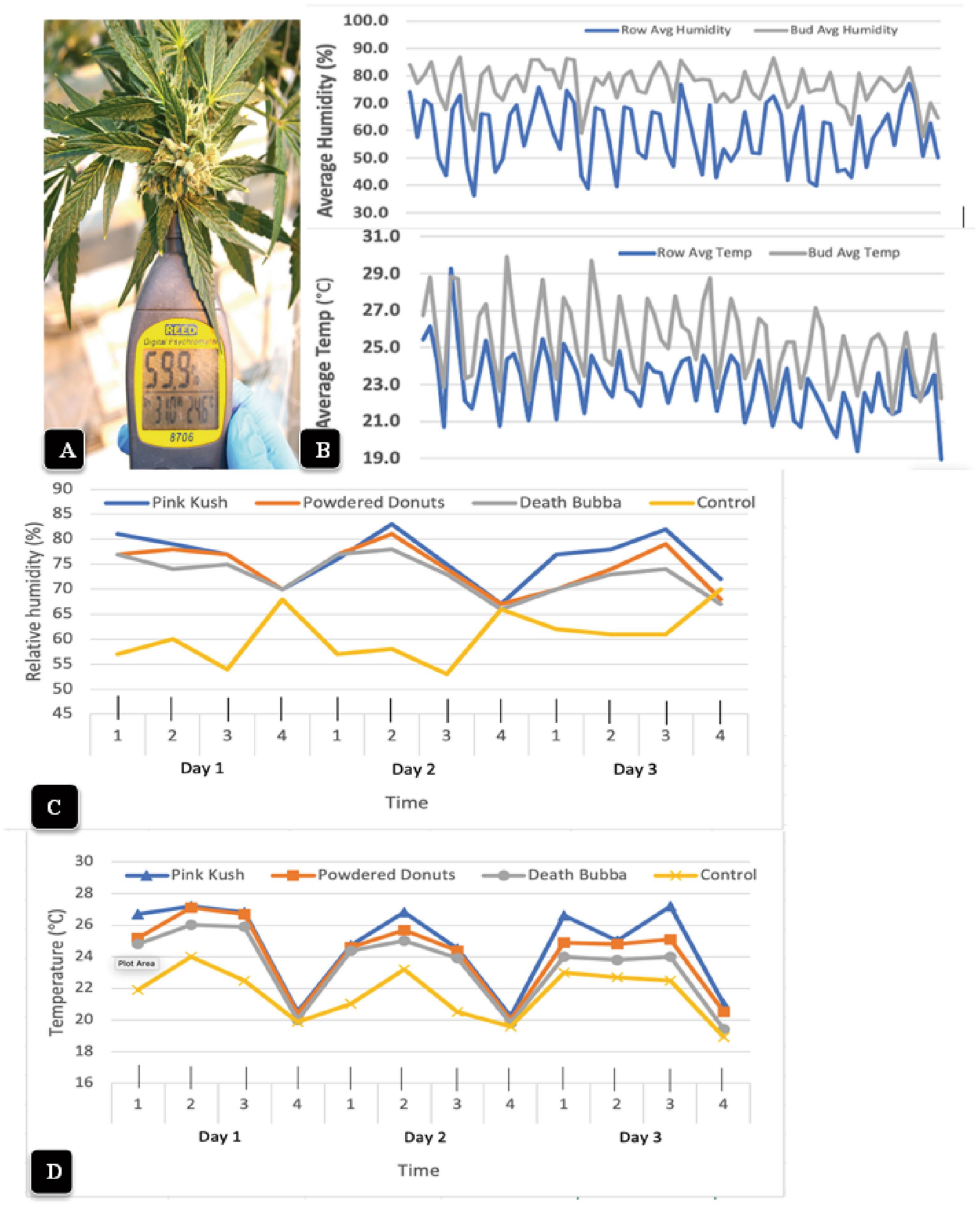
Measurement of microclimatic conditions within cannabis inflorescences **(A)** The hand-held Reed 8,706 psychrometer that was used to obtain measurements of temperature and relative humidity. The probe was inserted into the center of the inflorescence and held in place for 10 s. **(B)** Average relative humidity readings (top) and temperature readings (bottom) were taken over a 2-week period, four times a day, in the inflorescence (gray lines) compared to the ambient environment (blue lines) in the greenhouse. The measurements made from the inflorescence tissues (bud) of genotype “Pink Kush” were consistently higher at all time points compared to the ambient. **(C,D)** Measurements of relative humidity and temperature made over a 3-day period, four times a day, in inflorescences of four cannabis genotypes. The measurements made from the inflorescence tissues were consistently higher at all time points compared to the ambient control (yellow line). Note the daily fluctuations between day and night.

The psychrometer probe, when inserted into a cannabis inflorescence for 10 s (Figure 10A), provided readings of temperature and relative humidity that were measured four times a day over a 2 week period and compared to ambient conditions measured within the row of plants (Figure 10B). The humidity within the cannabis inflorescences averaged 75% (+/− 6%) over the duration of the observations, with the highest in the morning (6:45 a.m.) and lowest in the afternoon (2:45 pm) (Figure 10B). By comparison, the daily ambient relative humidity was much lower and the fluctuations were much greater, with an average of 55% (+/− 12%); pronounced dips to as low as 45% were observed at 2:45 p.m. With regard to temperature measurements within the inflorescence, the average was 26°C (+/− 3°C), with the highest in the afternoon (2:45 p.m.) and lowest in the morning (6:45 a.m.). The average ambient temperature over the same period of measurements was 23°C (+/− 3°C); pronounced dips to as low as 19.5°C were observed at 6:45 a.m. (Figure 10B). When the temperature and relative humidity measurements were repeated for two additional genotypes (“Powdered Donuts” and “Death Bubba”) in addition to “Pink Kush” over a 3-day period, the same trends were seen for all genotypes (Figure 10C). The relative humidity and temperature measurements for ambient conditions were always much lower compared to the inflorescences. While there were some differences among the three genotypes, they were not significant when compared to ambient conditions, which were much lower (Figure 10D).

**Figure 11:**
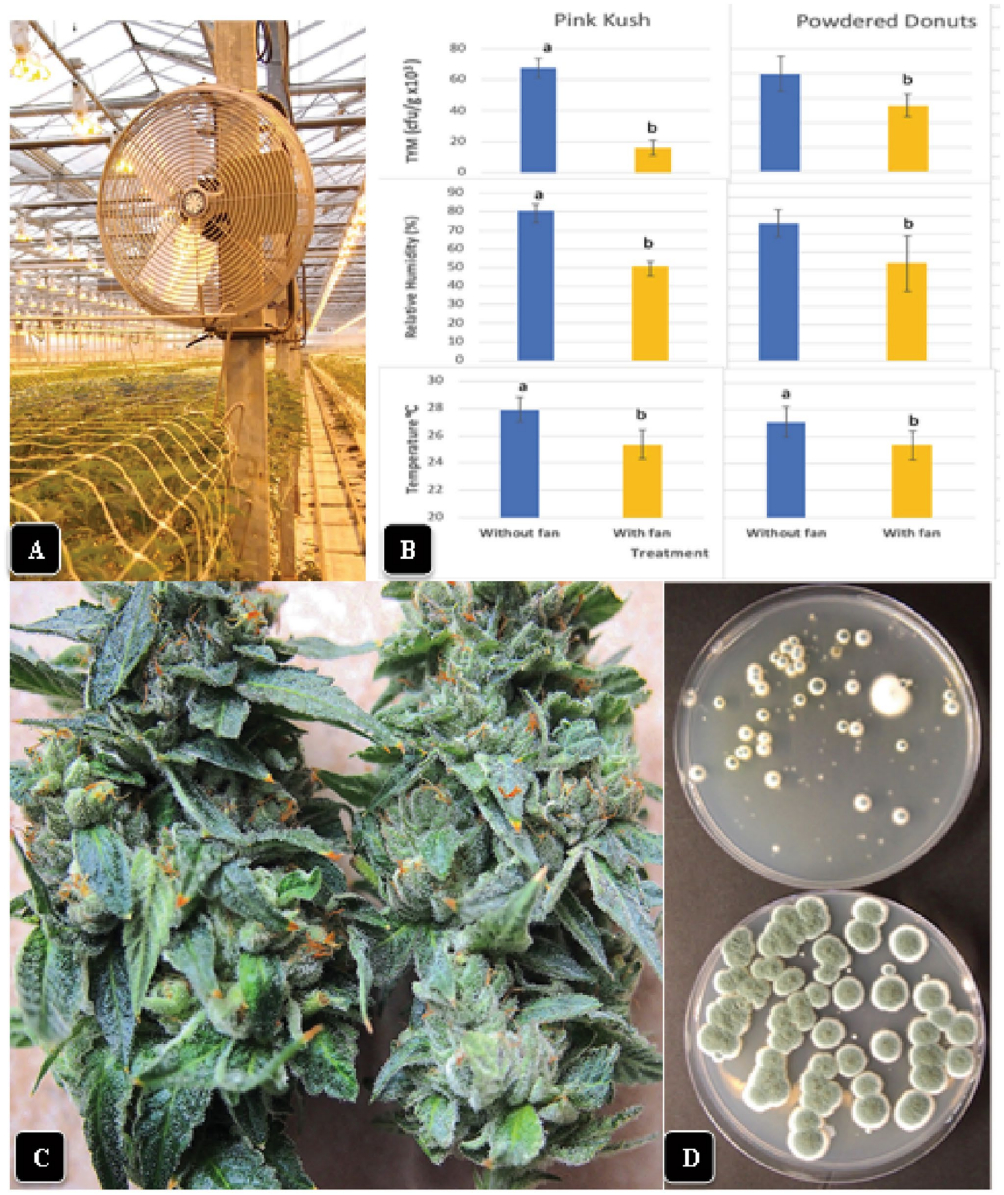
Effect of air circulation on TYM in cannabis inflorescences. **(A)** Placement of a fan that was used to circulate the air over cannabis plants for a 7-day period, 24 h per day. TYM levels were measured in inflorescences of treated and control plants without fans. **(B)** Comparison of temperature, relative humidity and TYM levels in inflorescences of two cannabis genotypes without and with fan treatment. Error bars show 95% confidence intervals and vertical bars with different letters are significantly different from each other according to Tukey’s *post-hoc* test (*p* < 0.05). **(C)** Samples of inflorescences from control (left) and treated (right) plants show a different appearance to the surface of the inflorescence leaves. The treated plants had a waxier appearance. **(D)** Recovery of TYM from inflorescences that were exposed to circulating air from fans (top Petri dish) compared to controls that did not receive air circulation (bottom Petri dish). The control dishes contain significantly higher levels of TYM compared to the treated.

The installation of fans over plants of “Powdered Donuts” and “Pink Kush” (Figure 11A) prior to harvest to provide air circulation for 24 h daily over a one-week period resulted in a significant (*p* < 0.05) reduction of TYM levels in inflorescences taken from treated compared to control plants (Figure 11B). Measurements of relative humidity and temperature in inflorescences of both sets of plants showed that they were both significantly (*p* < 0.05) reduced due to enhanced air circulation (Figure 11B). Inflorescences from plants with fans were observed to have a waxier, shinier surface that suggested enhanced cuticular deposition on the inflorescence leaves (Figure 11C); the effect on TYM colonies growing on agar medium can be seen in Figure 11D. Fewer colonies developed from inflorescences exposed to air circulation from fans compared to the controls without fans.

### 3.9. Effects of post-harvest handling methods on TYM levels

**Figure 12:**
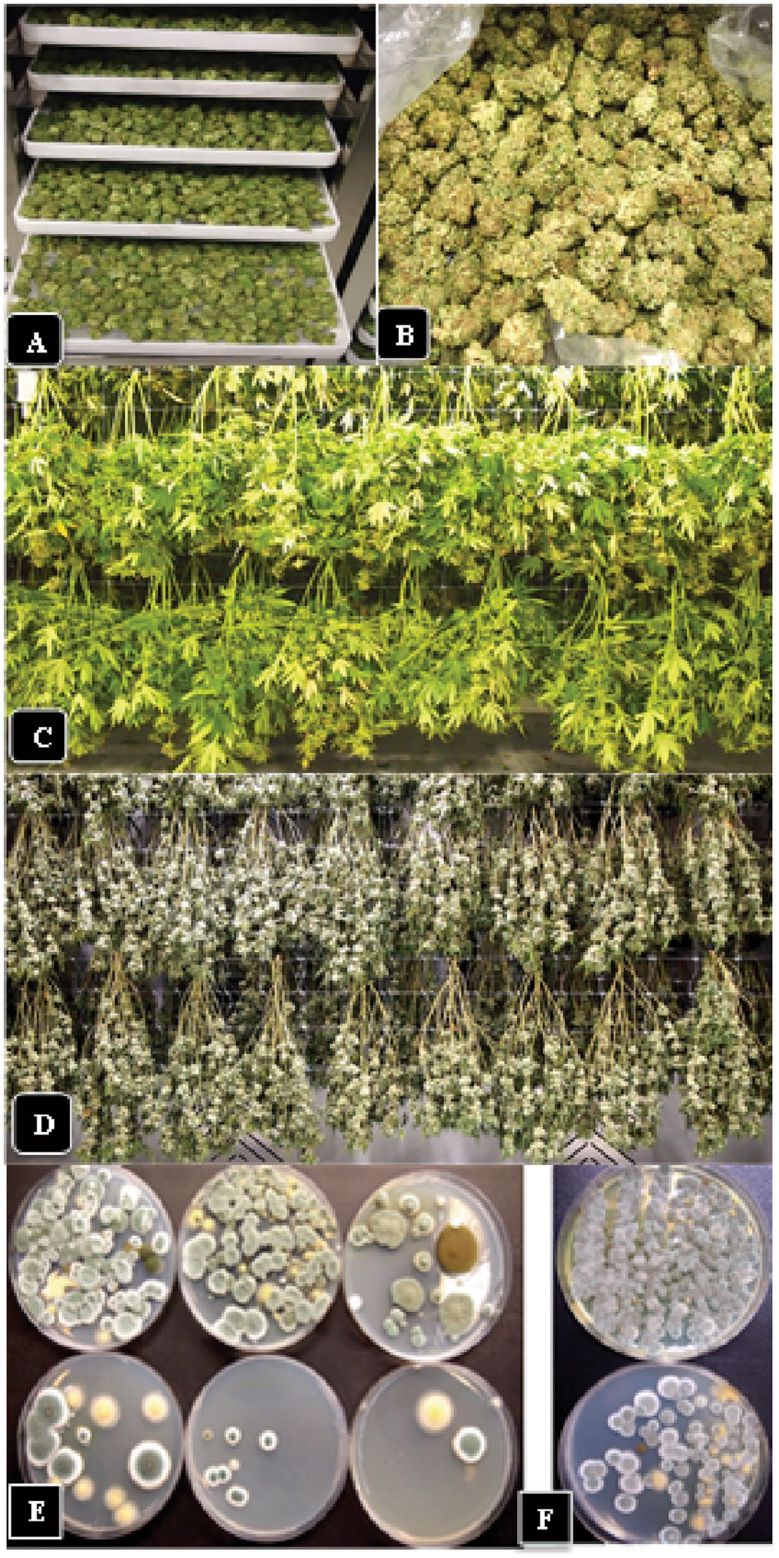
Effect of post-harvest handling methods on TYM levels. **(A,B)** The rack dry method involves removal of individual flowers (buds) from the stem followed by mechanical trimming of the inflorescence leaves (wet trim) and rack drying. **(C,D)** The hang-dry method involves drying the entire stem followed by removal of individual flowers from the stem and mechanical trimming of the inflorescence leaves (dry trim). **(E)** TYM levels present in three independent samples of inflorescences on day 1 in the drying room (top row of Petri dishes) compared to day 6 (bottom row of Petri dishes) following the hang-dry method. While the drying process significantly reduced TYM levels over 6 days, there were still remnants of microbes remaining, consisting mostly of *Aspergillus* and *Penicllium* spp. as seen on the Petri dishes. **(F)** Colonies of TYM observed on Petri dishes from the wet trim method (top Petri dish) compared to the hang dry method (bottom Petri dish). There were lower numbers of colonies recovered from the hang-dry sample.

Inflorescences from which individual buds were removed and subjected to a wet trim process to remove inflorescence leaves before being placed on drying racks (Figures 12A,B) were sampled for TYM analysis after 6 days of drying (Figure 12B). For comparison, inflorescences that were not removed and retained on the stem for a hang-dry method (Figures 12C,D), inclusive of a range of genotypes, were also analyzed.

**Figure 13:**
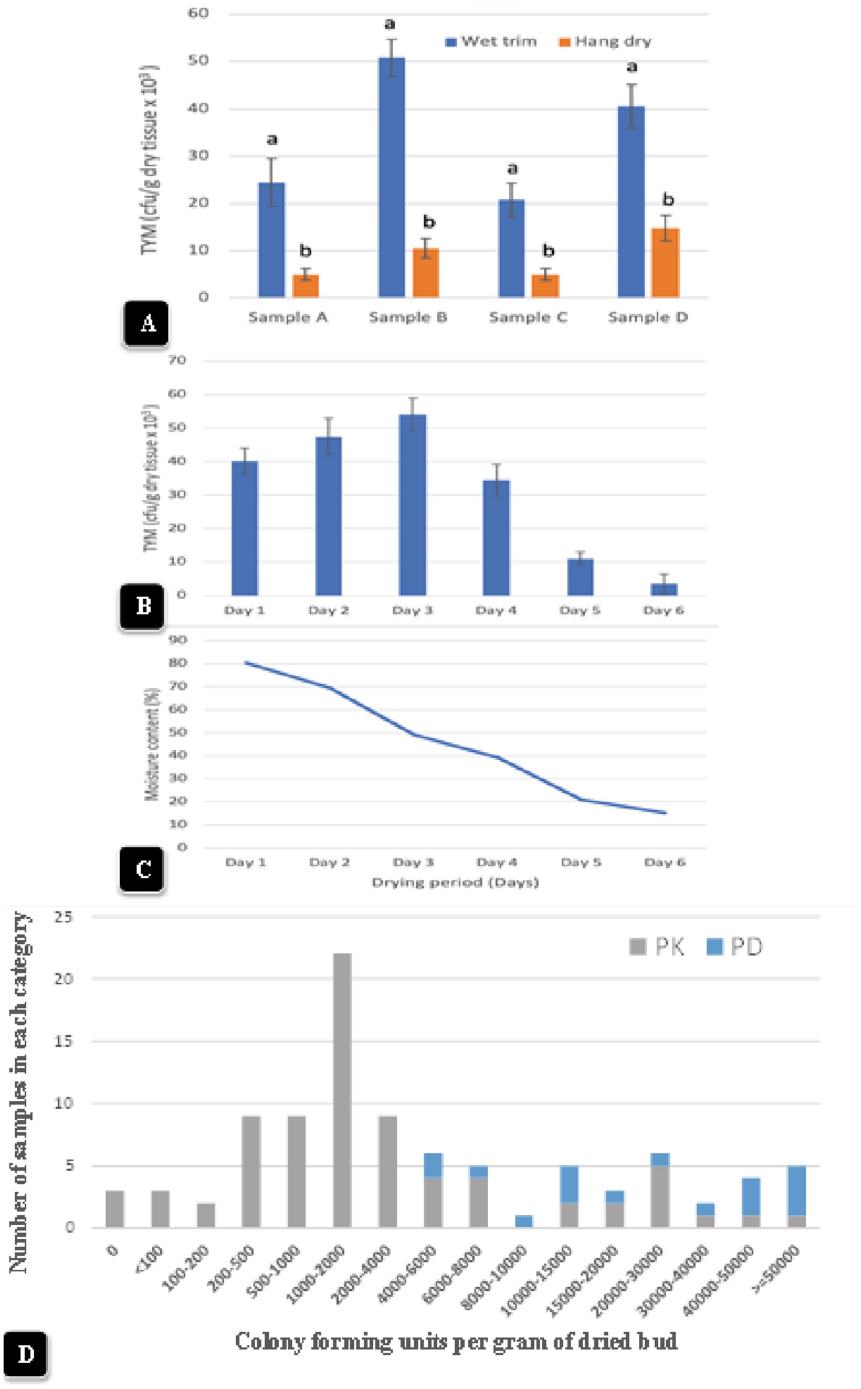
**(A)** The impact of wet-trim versus hang-dry method dry on TYM in four representative samples of cannabis. In every sample, the TYM levels were significantly higher in the wet trim compared to the hang dry method. Error bars show 95% confidence intervals and vertical bars with different letters are significantly different from each other according to Tukey’s *post-hoc* test (*p* < 0.05). **(B)** Changes in TYM over a 6-day period in hang dry cannabis inflorescences. On each sampling day, 12 random samples were obtained from the drying room and assayed for TYM levels. There was a rapid decline in TYM between days 4 and 6. The experiment was conducted three times with similar results. Standard error bars show 95% confidence intervals. **(C)** Change in moisture content in hang-dry cannabis samples after 6 days of drying. On each sampling day, 12 random samples were obtained from the drying room and assayed for moisture content and water activity. The final moisture content was 13% (with a range from 11 to14% in repeated samples). **(D)** TYM levels in two cannabis genotypes over a 12-month period of sampling grouped according to the number of samples that belonged to each TYM category. The hang-dry method was used and analysis of TYM was conducted by a commercial laboratory. The TYM levels in 110 samples ranged from <100 to >50,000 cfu/g. Samples of “Powdered Donuts” contained more overall TYM compared to “Pink Kush”, especially in the >50,000 cfu/g category.

Representative data from four samples are shown in Figure 13A. There was a significant (*p* < 0.05) reduction in TYM in all samples that were subjected to the hang-dry method compared to the wet-trim method. The appearance of colonies on PDA + S dishes after 5 days of incubation at 23°C is shown in Figures 12E,F. In all four samples, the number of colonies growing on dishes receiving homogenized extracts from wet trim samples was significantly greater compared to those receiving extracts from hang-dry samples. Most of the colonies were identified as *Penicillium*, *Aspergillus* and *Cladosporium*. The effect of the drying process on daily TYM levels in hang-dry inflorescences over a 6-day period is shown in Figure 13B. Following an initial increase in TYM levels during the first 3 days of drying, the populations dropped significantly to around 2,000 cfu/g on day 6. In none of the 35 samples tested was the TYM levels found to be at zero (data not shown). The impact of the drying process on the moisture content of 12 samples taken at daily intervals over 6 days from hang-dry inflorescences is shown in Figure 13C. There was a gradual but significant reduction in moisture content to an average of 12–14% (*n* = 48) after 6 days.

Inflorescence samples of genotype “Pink Kush” and “Powdered Donuts” processed using the hang dry method were sent to a commercial laboratory for estimation of TYM at various time points over a 12-month period from October 2020 to September 2021. A total of 110 samples were grouped according to the reported TYM levels. The data are presented according to the frequency of samples present in each category. The data in Figure 13D shows the TYM levels ranged from <100 to >50,000 cfu/g. The largest number of samples had TYM levels of 1,000–2,000 cfu/g. Only three samples out of 110 showed a TYM level close to zero, while 5 samples had TYM levels >50,000 cfu/g. When comparing genotypes “Pink Kush” and “Powdered Donuts” for TYM levels over a 12-month period, the latter genotype generally yielded samples with higher TYM levels compared to the former genotype (Figure 13D).

## 4. Discussion

The results from this study demonstrate for the first time that total yeast and mold (TYM) levels in cannabis inflorescences are significantly influenced by the genotype (strain) that is cultivated. The differences in TYM observed among six genotypes were consistent from replicated and repeated studies. Phenotypically, these genotypes differed in overall appearance, in growth rate and height, in total numbers, size, and density of inflorescences produced, and density of stigmas and trichomes (data not shown). Cannabis genotypes are known to differ significantly in the levels of cannabinoids and terpenes produced (Schwabe and McGlaughlin, 2019; Zager et al., 2019), and display different organoleptic properties in the final product that reflect these differences in chemical composition (chemotype). While this is the first report demonstrating that genotypic differences can affect microbial levels within cannabis inflorescences, in other plant species, phytobiome composition has been reported to be affected by host genetic composition (Whipps et al., 2008; Qian et al., 2018; Singer et al., 2021). As stated by Singer et al. (2021): “genetic differences within a given plant species can affect microbe recruitment, community assembly, and ultimately the composition of phytobiomes. As such, the phytobiome can be considered an extended phenotype of the plant that is determined by host genetics, the environment, and their complex interaction.” Our results highlight the importance of genotypic differences among cannabis plants which can influence the extent of microbial colonization of inflorescences.

The density of inflorescence leaves on different cannabis genotypes, which collectively created a warmer and more humid microclimate within the inflorescences, was shown to enhance TYM levels. This is supported by three lines of evidence. First, relative humidity and temperature measurements made within the inflorescences of three genotypes showed they were significantly higher compared to the ambient environment, pointing to the importance of the microclimate created by these leaves. Second, a negative impact on TYM populations was observed by enhanced air circulation using fans blowing over the plants, which reduced both the temperature and relative humidity within the inflorescences. This drier environment created by enhanced air circulation was observed to reduce microbial growth. Third, the overall levels of TYM in cannabis samples were consistently lower following post-harvest drying compared to fresh samples, reflecting the importance of moisture in enhancing TYM growth and survival. In hop flowers, the drying process was shown to reduce the overall microbial populations by up to 75% (Allen et al., 2019). Inflorescence leaves are metabolically active and contain abundant glandular trichomes and can manufacture up to 50% of the THC and CBD found in the inflorescence (Bernstein et al., 2019). The density of inflorescence leaves differed significantly among four cannabis genotypes studied, and a proportion of the TYM was found to reside in the inflorescence leaves themselves, even after surface-sterilization, suggesting they were internalized as endophytes. However, the majority of TYM resided as epiphytes, and were recovered by homogenizing tissues briefly before plating. The predominant fungi recovered were *Penicillium*, *Aspergillus*, and *Cladosporium* species, which are commonly present in the greenhouse environment (Punja et al., 2019).

The importance of trichomes, which are produced in abundance on bracts and inflorescence leaves on cannabis inflorescences (Punja et al., 2023), in supporting these microbial communities and/or permitting ingress by plant pathogens, has not been determined. On tomato leaves, trichomes produced on two different genotypes supported specific trichome-associated bacterial communities (Kusstatscher et al., 2007). In other plant species, trichomes served as habitats for microbes and potentially also provided sites for infection by plant pathogens (Kim, 2019). In addition to these interactions, trichomes potentially also provided sites for establishment of endophytic relationships with fungi (Kim, 2019). These types of trichome-microbe interactions have not been studied within cannabis inflorescences and can provide a better understanding of how these microbial populations become established. The ecological role of these microbes in potentially providing protection to the inflorescences from invasion by other fungi through competition was demonstrated through *in vitro* antagonism tests against several plant pathogenic fungi in this study (Supplementary Figure 2). Flowers of many plant species naturally contain a range of fungal, bacterial and yeast species with important ecological roles, including exerting competition against other microbes (Berg et al., 2017; Gouka et al., 2022). For example, a common cannabis epiphyte and endophyte, *P. olsonii* (Punja et al., 2019; Punja, 2021b), was recently demonstrated to colonize wheat spikes and induce transient expression of plant defense genes, which resulted in a reduction of Fusarium head blight development caused by *F. graminearum* (Rojas et al., 2022). Any potentially positive attributes of microbial colonizers of cannabis inflorescences deserve further attention.

Another feature of cannabis plants that can promote a build-up of TYM within inflorescences is the density of pistils (consisting of stigma, style and ovary tissues) and larger sizes of inflorescences observed in certain genotypes, such as “Watermelon Kush” and “Powdered Donuts”, that harbored consistently high TYM levels. Scanning electron microscopic observations showed that mycelium ramified over the pistils (in particular the stigmatic tissues) as well as the trichomes (Supplementary Figure 3). Pistils can provide sites on which bacterial and fungal populations can establish within flowers (Gschwend et al., 2021). A microbiome study of apple flowers, focused on the stigmatic tissues, showed that a diverse bacterial community was present, which could exert antagonistic activity against apple pathogens (Cui et al., 2021). In other plant species, such as pepper and wheat, stigmatic tissues provided infection sites for pathogenic fungi (Yang et al., 2010; Reis et al., 2016). A number of *Fusarium* species are reported to infect cannabis through a similar point of entry (Punja, 2021c), indicating the importance of stigmatic tissues in providing access to pathogens and TYM. Microbiome studies of the inflorescence tissues of cannabis should provide useful insights into the microbial communities, similar to what has been described from hop flowers (anthosphere microbiome) (Allen et al., 2019) and also in different tissue compartments within hemp plants (Barnett et al., 2020; Ahmed et al., 2021; Wei et al., 2021) and cannabis plants (Comeau et al., 2020). These studies would shed light on the temporal colonization of cannabis inflorescences as a function of genotype, environment and other factors, potentially leading to a better understanding of how TYM populations become established. Among the six genotypes included in this study, those that had a higher frequency of TYM levels in the inflorescences also appeared to show higher susceptibility to two pathogens of cannabis, namely powdery mildew and *Botrytis* bud rot. The range of levels of terpenes, THC or CBD among these genotypes did not correlate with a build-up of TYM.

Leaf litter left on the greenhouse floor after pruning activities harboured a range of fungi and yeasts, as shown by plating assays, the most prevalent of which were species of *Aspergillus*, *Cladosporium* and *Penicillium*. Leaves harbor a significant population of microbes at various stages of decomposition (Voříšková and Baldrian, 2013; Purahong et al., 2016). Harvesting activity, wherein workers remove foliage and prepare inflorescence stems for manual harvest, and step on the leaf litter, caused a significant increase in air-borne propagules of TYM, which were detected on air sampling plates. Cannabis inflorescences at harvest are sticky to the touch due to the secretion of resinous compounds from trichomes (Andre et al., 2016); spores of various fungi can adhere to the surfaces of stigmas and trichomes (Punja and Ni, 2021; Scott and Punja, 2022; Punja and Scott, 2023) and may be recovered on PDA + S medium. Subsequently, higher propagules of airborne fungi and yeasts can lead to higher TYM contamination. More fungal propagules would be expected to be present during the summer months compared to winter months (Punja et al., 2019); this could in part explain the higher incidence of TYM in samples collected during May–September compared to October–April in this study. A larger number of samples exceeded the 50,000 cfu/g limit in the former period compared to the latter.

In comparing PDA + S to Sabouraud dextrose and tryptic soy agars in this study, colonies on PDA + S were morphologically distinct and easier to identify and enumerate. McKernan et al. (2021) reported that potato dextrose agar amended with chloramphenicol provided the highest numbers and greatest diversity of fungal colonies from cannabis tissue samples compared to other agar media. Incubation of Petri dishes at 23°C provided more consistent colony size in the present study compared to the faster growing, and hence larger, intermingling colonies on dishes incubated at 28°C. An incubation period of 5 days under 10–14 h light was required to ensure colonies had reached a size that could be counted. Repay (2021) observed consistent and high recovery of TYM on Petrifilm™ medium. Schumacher et al. (2022) observed greater morphological diversity of colonies when dishes were incubated at 25°C compared to 28°C, confirming that temperatures of 22–25°C are more suited for TYM growth.

**Figure 14:**
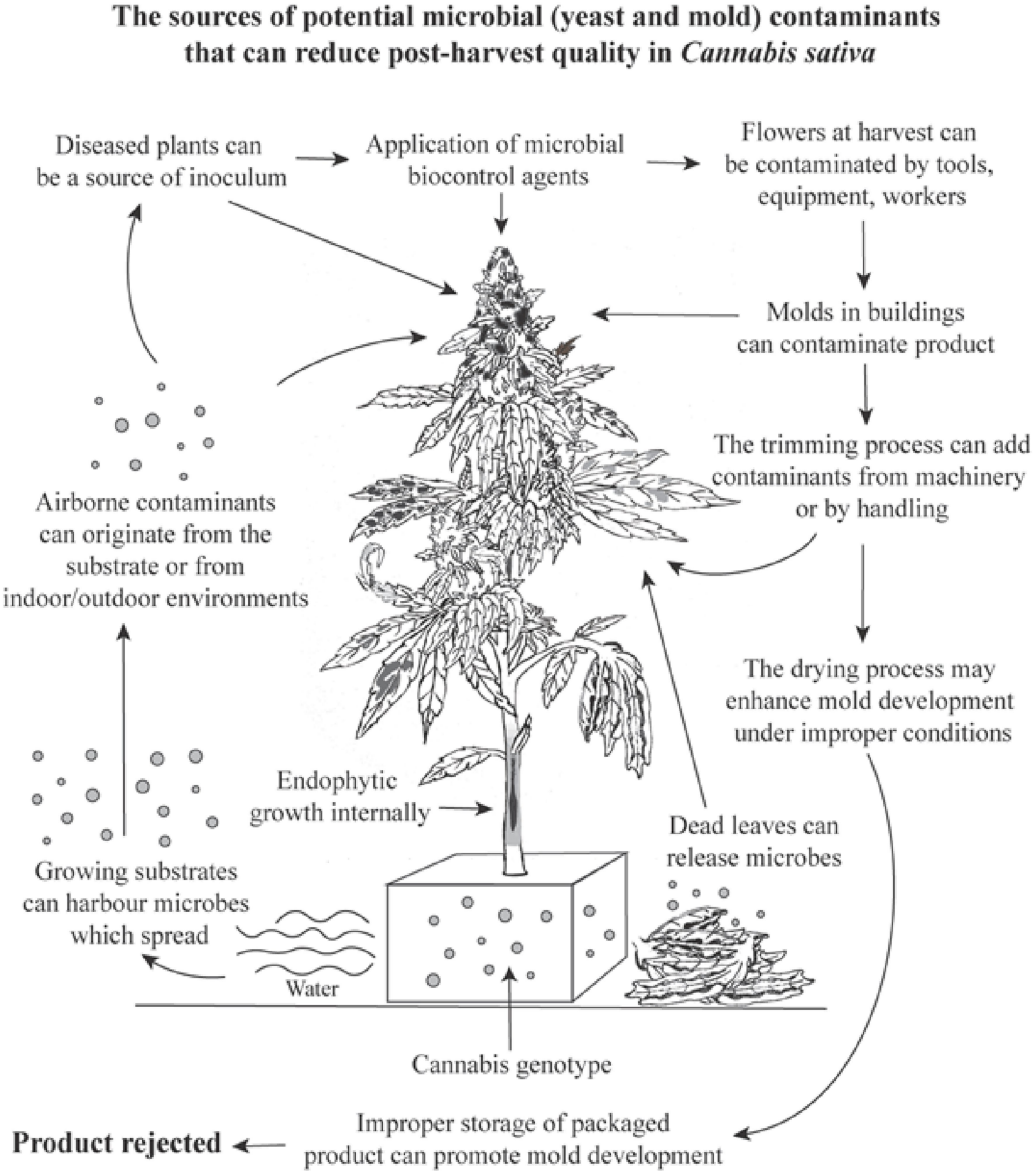
A summary of the potential variables and factors which can cause a build-up of total yeasts and molds in cannabis inflorescences, many of which were confirmed in this study. The overall increase in TYM levels can cause a product to fail to pass regulatory limits set by specific jurisdictions and cause the product to be rejected for sale.

Assessments of TYM in cannabis samples conducted by commercial laboratories may deploy a wide range of methods (Curry, 2022), which were not evaluated in our study. We instead focused on aspects of cannabis production methods that could affect TYM build-up during growth. When compared to the results for the same samples sent to two commercial laboratories, our method showed colony counts that were 7–22% greater than reported from one laboratory (lab A) and significantly higher (by up to 76%) than from a second laboratory (lab B) (Supplementary Table 1). The methods used by these laboratories are unknown, but lab B results may have greatly under-represented actual TYM levels. We homogenized samples, without pre-incubation, briefly (30 s) without the addition of buffers or reagents (in water) and rated colonies after 5 days at 23–25°C and 10–14 h light. These conditions may allow for a greater recovery of TYM compared to other approaches, but further research is needed to identify such variables. Discrepancies between commercial laboratories in TYM test results can be concerning from a regulatory perspective, since some cannabis samples reaching the consumer may contain higher levels of TYM than actually reported, depending on the laboratory performing the testing (Pusiak et al., 2021; Curry, 2022). The need for a universally adopted standardized method for TYM testing in the cannabis industry has been recognized (Curry, 2022; Jameson et al., 2022). Utilizing molecular-based qPCR analysis can identify specific subsets of TYM of concern to human health (Leppanen et al., 2019; McKernan et al., 2021). At present, all fungi and yeasts present in a cannabis sample, including those considered to be beneficial and approved for use during production by regulatory agencies and deemed to be safe for humans, are included in TYM assessment. For example, the application of biocontrol fungi, such as *Trichoderma* and *Gliocladium,* during cannabis production can increase TYM at harvest (Supplementary Figure 4), potentially causing them to fail under current regulatory requirements. A summary of the factors that can impact microbial presence in cannabis inflorescences is shown in Figure 14.

In addition to fungi, four yeast genera were recovered in this study – *Pseudozyma, Rhodotorula, Meyerozyma* (*Pichi*a) and *Candida* – which are widely distributed and present in various environments and on a range of plant species (Fernandez et al., 2012; Wirth and Goldani, 2012; Gafni et al., 2015; Gouka et al., 2022). They have not been previously reported from cannabis inflorescences and may constitute a component of the anthosphere. Epiphytic yeasts utilize a wide variety of nutrient sources and colonize a range of plant surfaces (Gafni et al., 2015). For example, *P. aphidis* (syn. *Moesziomyces aphidis)* (Kruse et al., 2017) was isolated from aphid secretions, produces extracellular polysaccharides and lipids (Lorenz et al., 2014) and occupies the same niches as pathogens such as powdery mildew (*Podosphaeria xanthii*) and gray mold (*Botrytis cinerea*) while secreting chitinase enzymes that contributed to its biocontrol activity on cucumber plants (Gafni et al., 2015). *Rhodotorula mucilaginosa* produces the siderophore rhodotorulic acid (Andersen et al., 2003), which showed *in vitro* suppressive effects against a number of fungi commonly found in cannabis inflorescences, including *Botrytis, Cladosporium, Penicillium* and *Rhizopus* (Calvente et al., 2001). However, *Rhodotorula* spp. have been considered as potential opportunistic yeast pathogens of humans under specific circumstances (Wirth and Goldani, 2012). *Pseuodozyma aphidis* demonstrated biological control activity against powdery mildew fungi (Gafni et al., 2015), potentially providing a benefit to the cannabis plant. Similarly, *Tilletiopsis* spp. present in hemp inflorescences were suggested to provide resident biocontrol activity (Barnett et al., 2020). *Pichia* and *Candida* species were recovered at a much lower frequency from cannabis inflorescences in this study and their potential importance remains undetermined.

Commercial drying of cannabis inflorescences is performed in specially designed drying rooms over a 5–7 day period at 21–23°C and 40–55% relative humidity (Caplan et al., 2022). These conditions ensure a slow removal of moisture to a final content of 12–14%, equating to a water activity of 0.65–0.7 (Caplan et al., 2022). Two general methods for handling cannabis inflorescences post-harvest are: (i) rack (screen) drying, where individual buds are mechanically removed from the stem followed by mechanical trimming of the inflorescence leaves (wet trim) and then dried, and (ii) hang-dry, where entire stems are hung upside down with leaves attached followed by trimming the inflorescence leaves after drying (Caplan et al., 2022). In a previous study (Punja et al., 2019), wet trim was reported to cause injury to inflorescence leaves that resulted in a significant build-up of microbial populations post-harvest. In the present study, a comparison of wet trim and hang dry processes showed that TYM in samples processed using the hang-dry method were significantly lower. The absence of injury sites for colonization by resident or introduced microbes in trimmed dried samples compared to trimmed fresh samples would account for the difference between the two handling methods.

During the hang dry process, the foliage present on the stems (see Figures 12C,D) caused a spike in TYM during the first 72 h. The air in the drying rooms also showed a significant increase in the population of air-borne propagules, likely to have originated from the leaf tissues (data not shown). Leaves can harbour a significant population of microbes at various stages of decomposition (Voříšková and Baldrian, 2013; Purahong et al., 2016). Upon further drying to 96 h, the TYM levels were significantly reduced as the tissue moisture content was reduced to 12–14%. During moisture removal, microbial populations rapidly decline since drier conditions are unfavorable for growth of most yeasts and molds, which occurs optimally at water activities (a_w_) near 0.9 (Beuchat, 1983). The minimum a_w_ at which growth of fungi has been observed is around 0.61 (Beuchat, 1983). However, certain species of yeasts and fungi, can grow and survive at a _w_ of 0.62–0.7. These xerotolerent microbes, which include species of *Aspergillus* and *Penicillium*, were commonly present in cannabis inflorescences in this study. Cannabis products with a water activity >0.70 a_w_ but less than 0.86 a_w_ are considered shelf-stable but will still support the growth of molds and yeasts (Carter, 2019). Our data showed that even after 6 days of drying to a moisture content of 12–14% (translating to a_w_ of *ca.* 0.62–0.7), there were still remnants of microbial activity that could be detected on plating media. Inadequate drying of cannabis samples that leave pockets of moisture can result in a higher survival rate of TYM. In subsequent handling processes, such as curing, storage and shipping, a proliferation of these residual microbes could take place. As a step toward totally eliminating TYM in dried cannabis samples, the use of electron-beam irradiation was shown to reduce microbial activity in treated samples to undetectable levels (Punja, 2021b). While deemed to be an effective method to eliminate TYM in cannabis (Jerushalmi et al., 2020; Dhillon et al., 2022), it is a costly process and does not eliminate mycotoxins (Khaneghah et al., 2020; Akhila et al., 2021). Also, it is not approved for use in all jurisdictions and the impact on levels of cannabinoids and terpenes has not been well established.

The 12-month study quantifying the TYM levels in two cannabis genotypes (“Pink Kush” and “Powdered Donuts”) that were processed using the hang dry method showed that from 110 samples, the largest number contained TYM levels of 1,000–2,000 cfu/g. Only three samples had a TYM level close to zero, while 5 samples out of 110 had TYM levels >50,000 cfu/g. This indicates that the majority of greenhouse-grown cannabis tissues generally contain a low frequency of TYM, and the extent to which they may proliferate could be influenced by the genotype and/or post-harvest handling and storage conditions. In comparing “Pink Kush” and “Powdered Donuts” for TYM levels over the 12-month period, the latter genotype generally yielded more samples with higher TYM levels compared to the former genotype, confirming previous observations from this study on genotypic differences.

TYM testing of cannabis products follows protocols established from the food industry, where contamination by yeasts and molds can lead to severe food-borne illnesses and poor quality of product (Tournas et al., 2001; Boyar, 2021). Since cannabis is not directly ingested, but rather inhaled or vaped or further extracted and utilized, it seems reasonable that the presence of, and potential threat to humans, of specific microbes and their by-products, such as mycotoxins, should be assessed. For example, the two most prevalent species of *Aspergillus* observed in this study – *A. ochraceus* and *A. niger*—are both known to produce Ochratoxin A (OTA), in addition to *Penicillium* spp. OTA is a nephrotoxic and carcinogenic agent commonly found in a range of fungal-contaminated agricultural products (Moss, 2008; Pitt and Hocking 2009b; Malir et al., 2016; Atumo, 2020). A number of jurisdictions, including the state of California, require mandatory testing for four *Aspergillus* spp. (Boyar, 2021; Jameson et al., 2022). Yet, surprisingly, *A. ochraceus*, the most commonly recovered fungus from inflorescences and leaves of greenhouse-grown cannabis in the present study, is not mentioned in previous reports of fungi of concern to human health present in cannabis tissues (Leppanen et al., 2019; Boyar, 2021; McKernan et al., 2021). This species, as well as *A. niger*, are widely distributed, and are present in dried foods, including dried fish, various dried beans and pulses, nuts and oilseeds (Pitt and Hocking, 1997; Pitt and Hocking, 2009a). The presence of both species in grapes and green coffee beans may lead to ochratoxin A production (Mantle and Chow, 2000; Perrone et al., 2007; Pitt and Hocking, 2009a; Pandit et al., 2014). Neither species is currently included for specific testing in cannabis, nor are *Fusarium* spp., although these fungi are known to produce a range of mycotoxins and both are frequently present in cannabis inflorescences (Punja, 2021c; Gwinn et al., 2022). In addition, up to 34 different fungal species can be recovered from dried cannabis samples in commercial facilities to varying degrees (Punja, 2021b). Mycotoxin production is affected by water activity, and generally fungal growth at a_w_ of 0.83–0.9 is required (Beuchat, 1983). Most rapid colonization of cannabis inflorescences by potential mycotoxin-producing fungi would likely occur during inflorescence growth prior to harvest (Punja, 2021c) and subsequent drying to a_w_ values of 0.62–0.7 would not impact any mycotoxin accumulation that has already occurred. This highlights the importance of pre-harvest monitoring and management of inflorescence-contaminating fungi to reduce their potential impact on cannabis consumers. A re-examination of the fungal and yeast species considered by regulatory agencies to pose a threat to consumers of cannabis would seem prudent. Many identified in this study are not considered harmful, yet would be included in current TYM testing regulations.

## 5. Conclusion

Our results highlight the various factors which can cause a build-up of TYM to occur in inflorescences during cannabis production. We have also identified some of the variables that can potentially cause samples to fail to meet regulatory standards due to excessive TYM. However, different cfu/g limits set by different jurisdictions make it challenging for cannabis producers. In different states within the USA, for example, acceptable levels of TYM differ significantly.^4^ In countries such as Canada, where cannabis is approved for medicinal and recreational use, the guidelines are uniform (Health Canada, 2019; Pusiak et al., 2021). The inconsistencies in cannabis product testing between different laboratories raises an ongoing concern, and the development of standardized methods for quantifying TYM needs to be addressed by regulatory agencies to ensure there is consistent product quality for the consumer, as stated by Jameson et al. (2022). Not all species of TYM present in cannabis inflorescences pose potential harm to humans; some have been demonstrated to have antifungal activity against deleterious plant pathogens of cannabis and may be exerting a natural form of biological control often reported in other plant species. Distinguishing between those yeasts and molds that are beneficial from those that are harmful will continue to present a challenge, since the majority of current testing protocols rely solely on an analysis of total colony-forming units. Species-specific molecular testing can be one approach to address this issue. The results from this study identify avenues for producers to monitor and manage the potential build-up of TYM in fresh and dried cannabis samples that should lead to lower TYM and a higher quality product. This study has emphasized greenhouse grown cannabis. Other production systems, such as field-grown or organically grown cannabis, also need to be assessed, as there can be differences in the amount and types of TYM present (Punja and Scott, 2023).

## Data availability statement

The original contributions presented in the study are included in the article/Supplementary material, further inquiries can be directed to the corresponding author.

## Author contributions

ZP conducted the design of experiments, development of methods, collection of data, supervision of the project, preparation of figures, and writing of the manuscript. LN conducted the experiments on TYM plating. SL and LB conducted the collection of samples from the greenhouse and drying rooms and associated measurements and analysis. All authors contributed to editing of the manuscript and finalization of figures for publication.

## Funding

This research was jointly funded by an Alliance Grant from the Natural Sciences and Engineering Research Council of Canada (NSERC) (grant number 571270–21) and industry funding from Pure Sunfarms. Additional funding was provided by the B.C. Ministry of Agriculture/Agriculture and Agri-Food Canada through the Canadian Agricultural Partnership (CAP) Program Project No. URACP 19–212.

## Conflic of interest

The authors declare that the research was conducted in the absence of any commercial or financial relationships that could be construed as a potential conflict of interest.

## Publisher’s note

All claims expressed in this article are solely those of the authors and do not necessarily represent those of their affiliated organizations, or those of the publisher, the editors and the reviewers. Any product that may be evaluated in this article, or claim that may be made by its manufacturer, is not guaranteed or endorsed by the publisher.

## Footnotes

^1^ https://www.medicinalgenomics.com/cannabis-microbial-testing-regulations-by-state/

^2^ http://www.pharmacopeia.cn/v29240/usp29nf24s0_c731.html

^3^ https://www.gmp-compliance.org/gmp-news/new-usp-chapter-922-water-activity

^4^ https://www.medicinalgenomics.com/cannabis-microbial-testing-regulations-by-state/
