# Supplementary material for: Total yeast and mold levels in high THC-containing cannabis (Cannabis sativa L.) inflorescences are influenced by genotype, environment, and pre-and post-harvest handling practices
Source: Front Microbiol. 2023 Jun 13;14:1192035. doi: 10.3389/fmicb.2023.1192035 (PMC10294073; doi:10.3389/fmicb.2023.1192035)

**Supplementary Table 1.** Total yeast and mold levels (cfu/g) in fresh and dried cannabis flower and leaf samples as determined in this study and reported by two commercial laboratories.

______________________________________________________________________________

**Total yeast and mold levels (cfu/g)^a^**

Sample Present study Commercial Lab A Commercial Lab. B

______________________________________________________________________________

**Fresh flower 1** 2.5 x 10^5^  2.0 x 10^5^ (-20%) 0.6 x 10^5^ (-76%)

**Fresh flower 2** 2.0 x 10^5^  1.7 x 10^5^ (-15%) 0.41 x 10^5^ (-79%)

**Dried flower 1** 4.5 x 10^3^  4.2 x 10^3^ (- 7%) 1.9 x 10^3^ (-57%)

**Dried flower 2** 2.6 x 10^3^ 2.2 x 10^3^ (-15.4%) 0.9 x 10^3^ (-65%)

**Leaf litter (fresh)** 3.5 x 10^4^  2.7 x 10^4^ (-22.8%) 1.6 x 10^4^ (-54%)

**Leaf litter (dried)** 2.2 x 10^4^  1.6 x 10^4^ (-27%) 0.7 x 10^4^ (-68%)

______________________________________________________________________________

^a^ Data are the results from an analysis of the same bulk tissue sample, which was divided into 3 sub-samples, and sent to 2 different laboratories for TYM levels. One sub-sample was plated on PDA+S as described in this study. Values in brackets show the deviations in values from the present study.

**SUPPLEMENTARY FIGURE 1**

Recovery of yeast species originating from cannabis inflorescences after grinding and plating tissues onto PDA+S. **(A)** Small pink colonies of *Moesziomyces (Pseudozyma) aphidis.* **(B)** Colonies of *Moesziomyces aphidis* growing out of cannabis leaf segments after 3 weeks of incubation. **(C)** *Pseudozyma flocculosa* from a streak culture showing light pink mycelial growth. **(D)** *Meyerozyma* (*Pichia*) *guilliermondii* from a streak culture showing white mycelial growth. **(E)** Reddish-orange colonies of *Rhodotorula mucilaginosa.* **(F)** Bright yellow colonies of the bacterial species *Novosphingobium capsulatum* were recovered from cannabis tissues. **(G)** The genera and species of fungi and yeasts recovered from cannabis inflorescences in this study and the corresponding GenBank **
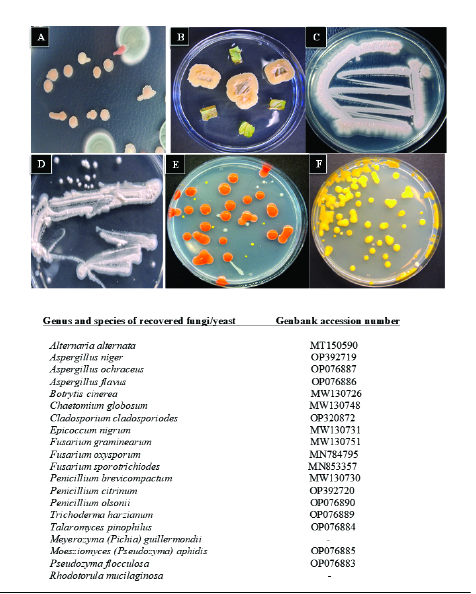
**accession numbers for sequences of the ITS—ITS2 region of rDNA.


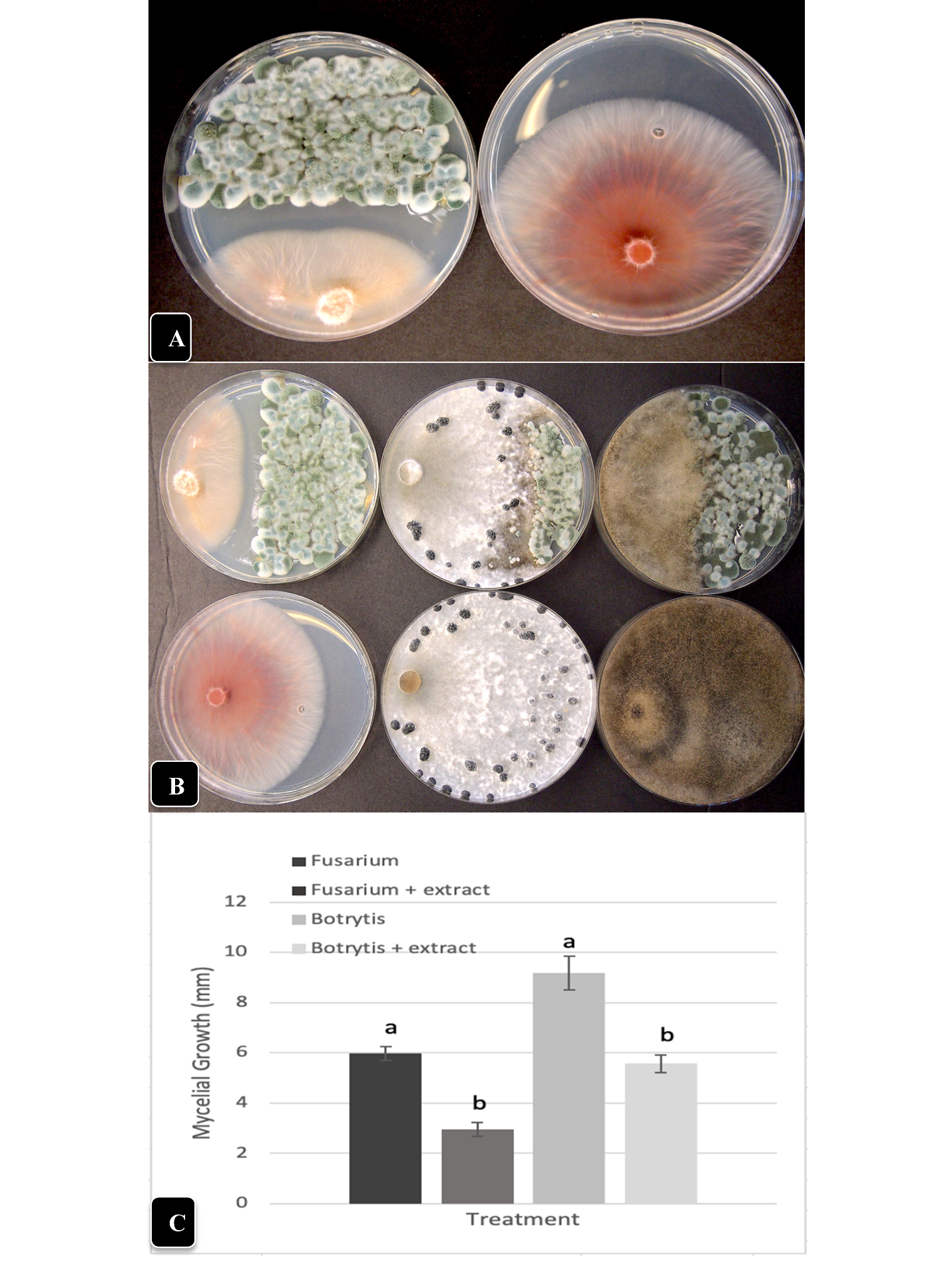
**SUPPLEMENTARY FIGURE 2**

**(A)** The morphology of an inflorescence of ‘Watermelon Kush’ in which the inflorescence leaves have been dissected to show the abundance of orange-brown stigmas covering the surface. **(B)** Natural infection of ‘Watermelon Kush’ by a *Fusarium* sp. showing the pinkish-white mycelium colonizing the stigmas and growing through the flower tissues. **(C)** Recovery of pinkish-red colonies from plated stigmatic tissues on PDA+S reveals the presence of *F. sporotrichiodes****.* (D, E)** Scanning electron microscopic images of the inflorescence tissues from **(B)** shows mycelium (arrows) of *Fusarium* which has grown over tissues bearing glandular trichomes **(D)** as well as over the stigmatic tissues. **(E).** The mycelial growth has covered the entire inflorescence tissues including the stigmas (S).

**SUPPLEMENTARY FIGURE 3**


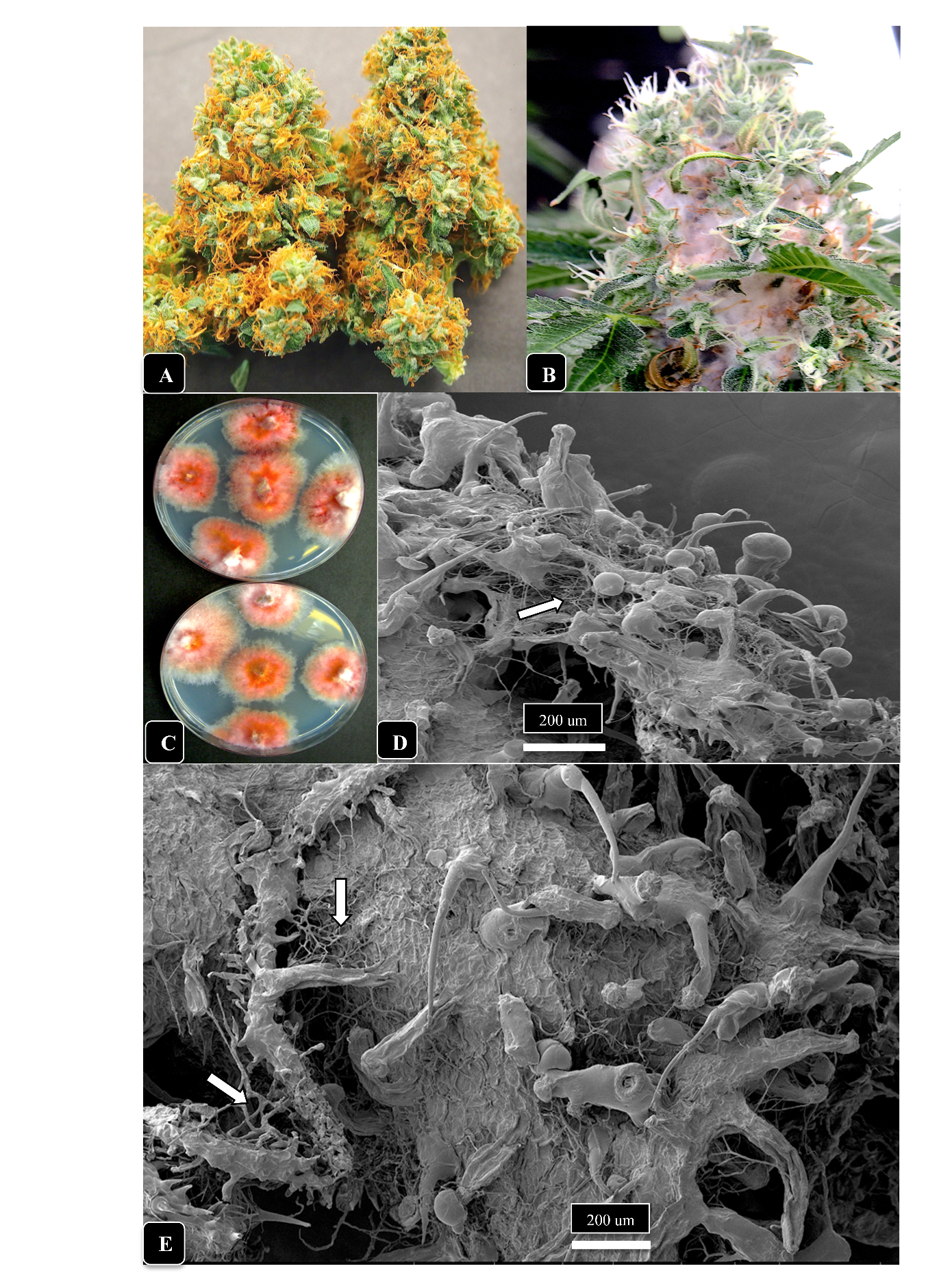


The inhibitory effect of blended inflorescence tissues containing TYM on growth of fungal pathogens in vitro. **(A)** The Petri dish on the left contains a swab made from blended inflorescence tissue (IN) while the dish on the right is the control. In both dishes, a mycelial plug of *Fusarium oxysporum* was placed 48 hr earlier at the far end of the dish. The inhibition of colony growth can be seen with the IN but not in the control**. (B)** Inhibition of growth by IN against 3 fungal pathogens. On the left is *F. oxysporum*, in the middle is *Sclerotinia sclerotiorum*, and far right is *Botrytis cinerea*. All 3 fungi were inhibited to varying degrees by the IN. **(C)** Linear growth of mycelium of 2 fungal pathogens (*Fusarium* and *Botrytis*) in the absence or presence of blended inflorescence tissues from cannabis. Error bars show 95% confidence intervals and vertical bars with different letters are significantly different from each other according to Tukey’s post-hoc test (p < 0.05).

**SUPPLEMENTARY FIGURE 4**

The effect of applications of two registered biological control products on TYM levels in dried cannabis inflorescences at harvest. The products were Prestop WP (1 g/L, containing *Gliocladium catenulatum* at 1 x10^9^ colony-forming units/g) and Rootshield HC (10 g/L, containing *Trichoderma harzianum* at 1x10^7^ colony-forming units/g). Applications to flowering plants of ‘Pink Kush’ were made at 14, 21 and 28 days into the flowering period for Rootshield HC and once at 21 days into the flowering period for Prestop (due to label restrictions). Inflorescences from treated and adjoining rows of untreated plants were harvested 4 weeks after the final spray (week 8 of flower development) and hang-dried, and then sent to a commercial laboratory for TYM analysis. **(A)** TYM levels in dried cannabis inflorescences from four greenhouse trails. In trial 3, applications of Rootshield caused the TYM levels to exceed the 50,000 cfu/g limit. In the other trials, TYM levels were increased by the biocontrol treatments. **(B)** Growth of colonies of *G. catenulatum* (left) and *T. harzianum* (right) on PDA+S from dried cannabis samples taken following applications of the respective biocontrol agents. **(C)** Colonies of *Penicillium* (left) from untreated cannabis inflorescences compared to those receiving *T. harzianum* showing reduced *Penicillium* colonies (right) from treated buds at harvest.


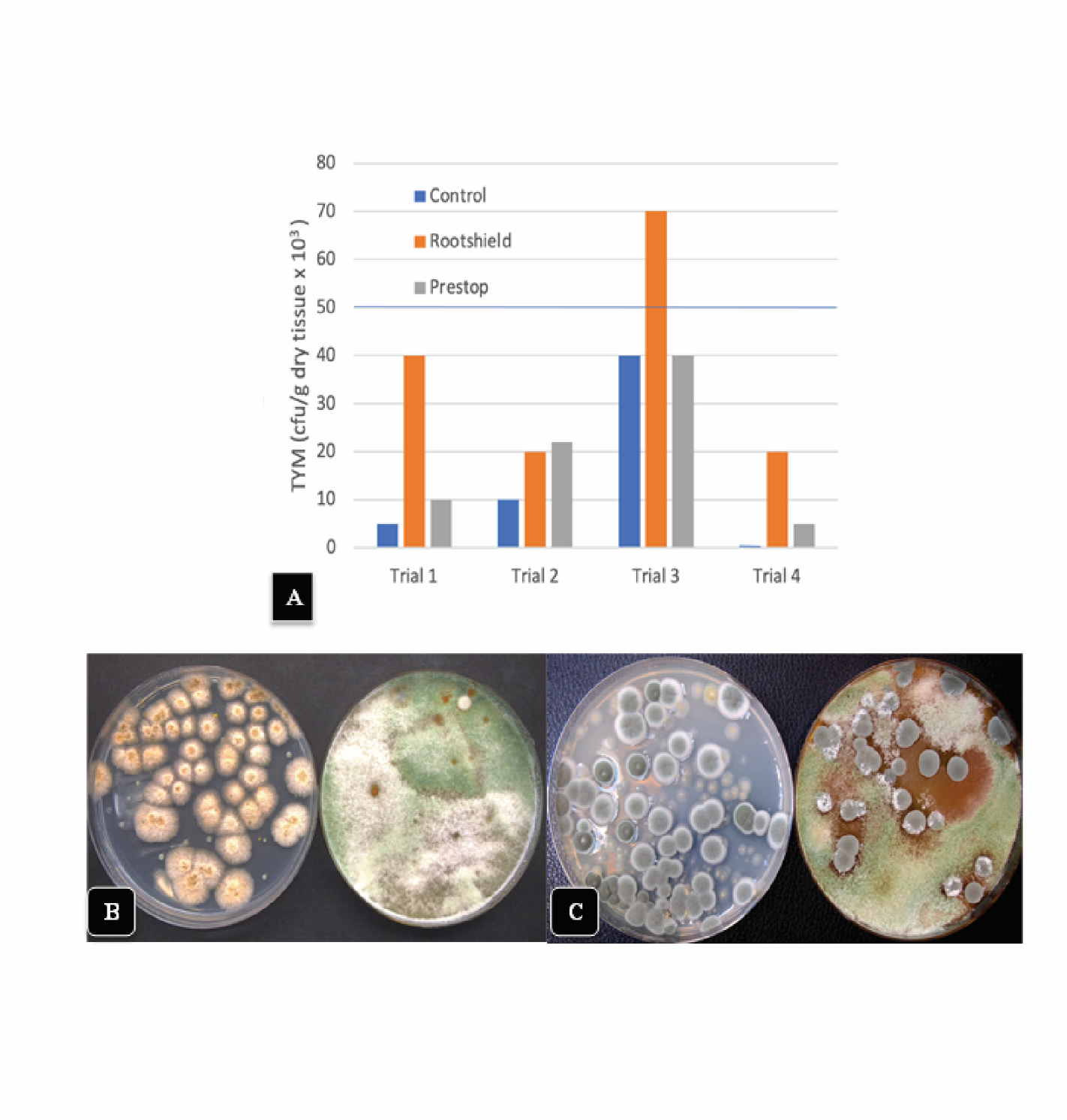

Supplement: Supplementary file 1 [file Data_Sheet_1.docx]
